# CPT1A-mediated fatty acid oxidation promotes cell proliferation via nucleoside metabolism in nasopharyngeal carcinoma

**DOI:** 10.1038/s41419-022-04730-y

**Published:** 2022-04-11

**Authors:** Min Tang, Xin Dong, Lanbo Xiao, Zheqiong Tan, Xiangjian Luo, Lifang Yang, Wei Li, Feng Shi, Yueshuo Li, Lin Zhao, Na Liu, Qianqian Du, Longlong Xie, Jianmin Hu, Xinxian Weng, Jia Fan, Jian Zhou, Qiang Gao, Weizhong Wu, Xin Zhang, Weihua Liao, Ann M. Bode, Ya Cao

**Affiliations:** 1 Key Laboratory of Carcinogenesis and Cancer Invasion, Chinese Ministry of Education, Department of Radiology, Xiangya Hospital, Central South University, 410078 Changsha, China; 2 Cancer Research Institute and School of Basic Medical Science, Xiangya School of Medicine, Central South University, 410078 Changsha, China; 3 Key Laboratory of Carcinogenesis, Chinese Ministry of Health, 410078 Changsha, China; 4 Molecular Imaging Research Center of Central South University, 410008 Changsha, Hunan China; 5 Department of Laboratory, National Cancer Center / National Clinical Research Center for Cancer/Cancer Hospital, Chinese Academy of Medical Sciences and Peking Union Medical College, 100021 Beijing, China; 6 Key Laboratory for Carcinogenesis and Cancer Invasion, Chinese Ministry of Education, Zhongshan Hospital, Shanghai Medical School, Fudan University, 200000 Shanghai, China; 7 Department of Otolaryngology Head and Neck Surgery, Xiangya Hospital, Central South University, 410078 Changsha, China; 8 Department of Radiology, Xiangya Hospital, Central South University, 410078 Changsha, China; 9 The Hormel Institute, University of Minnesota, Austin, MN 55912 USA; 10 Research Center for Technologies of Nucleic Acid-Based Diagnostics and Therapeutics Hunan Province, 410078 Changsha, China; 11 National Joint Engineering Research Center for Genetic Diagnostics of Infectious Diseases and Cancer, 410078 Changsha, China; 12 National Clinical Research Center for Geriatric Disorders, Xiangya Hospital, Central South University, 410078 Changsha, China

**Keywords:** Cancer metabolism, Cell growth

## Abstract

As the first rate-limiting enzyme in fatty acid oxidation (FAO), CPT1 plays a significant role in metabolic adaptation in cancer pathogenesis. FAO provides an alternative energy supply for cancer cells and is required for cancer cell survival. Given the high proliferation rate of cancer cells, nucleotide synthesis gains prominence in rapidly proliferating cells. In the present study, we found that CPT1A is a determining factor for the abnormal activation of FAO in nasopharyngeal carcinoma (NPC) cells. CPT1A is highly expressed in NPC cells and biopsies. CPT1A dramatically affects the malignant phenotypes in NPC, including proliferation, anchorage-independent growth, and tumor formation ability in nude mice. Moreover, an increased level of CPT1A promotes core metabolic pathways to generate ATP, inducing equivalents and the main precursors for nucleotide biosynthesis. Knockdown of CPT1A markedly lowers the fraction of ^13^C-palmitate-derived carbons into pyrimidine. Periodic activation of CPT1A increases the content of nucleoside metabolic intermediates promoting cell cycle progression in NPC cells. Targeting CPT1A-mediated FAO hinders the cell cycle G1/S transition. Our work verified that CPT1A links FAO to cell cycle progression in NPC cellular proliferation, which supplements additional experimental evidence for developing a therapeutic mechanism based on manipulating lipid metabolism.

## Introduction

Metabolic reprogramming contributes to the competence of tumor cells in the modification of their metabolism to support the extended energy demand due to their rapid growth and proliferation [1]. Lipids are spatially and temporally regulated during cell division, support changes in the plasma membrane, and assist in the separation of membrane-bound organelles [2]. Lipid metabolism is altered in rapidly proliferating cells, such as cancer cells [3]. Abnormal fatty acid oxidation (FAO) is an important part of tumor energy metabolic reprogramming [4]. By shuttling long-chain fatty acids into mitochondria, carnitine palmitoyl transferase 1 (CPT1) constitutes a rate-limiting step of FAO. CPT1A is a subtype of the CPT1 transport system, controlling the entry of fatty acids into the mitochondria for oxidation [5]. The metabolic intermediate acetyl-CoA enters the Krebs cycle coupling of mitochondrial oxidative phosphorylation to produce adenosine triphosphate (ATP) and NADPH [6]. In mitochondrial FAO pathways, targeting CPT1A generates clinical benefits in radiation therapy for NPC and breast cancer patients [7, 8].

NPC is a major subtype of head and neck squamous cell carcinoma (HNSCC), with high incidence in Southeast Asia [9]. NPC incidence is closely related to Epstein-Barr virus (EBV) infection, genetic susceptibility, and environmental factors [10, 11]. In HNSCC, the frequency of chromosome 11q13 segment amplification is approximately 40%, and amplification is associated with the appearance of lymph node metastasis and poor prognosis [12]. Importantly, a high amplification frequency in the 11q13.1-13.3 region, where the *CPT1A* gene is located, is found in NPC. An increased copy number of *CPT1A* is associated with decreased overall survival in patients with esophageal squamous cell carcinoma [13].

During tumor progression, amplified metabolic genes may facilitate carcinogenic drivers through metabolic reprogramming, conferring tumor survival and growth advantages [14]. Cell cycle regulators participate in metabolic remodeling, whereas metabolic regulation promotes cell cycle progression, suggesting a bidirectional connection between cell division and general metabolism [15]. CPT1A-dependant FAO plays an essential role in the cell cycle progression of cancer cells. CPT1A may be a mechanistic link between lipid catabolism and cell cycle regulation.

Metabolic intermediates derived from glucose, glutamine, and aspartate are required for de novo nucleotide synthesis. The ready availability of a pool of nucleotides facilitates appropriate DNA repair and replication [16]. The glutamine synthetase catalyzation of glutamine mainly contributes to fuel de novo nucleotide synthesis and accelerate DNA repair [17]. The loss of CPTIA leads to inhibition of de novo nucleotide synthesis and DNA replication, which suggests that fatty acid carbons are essential for the synthesis of dNTPs in endothelial cells [18]. Given the high proliferation rate of cancer cells and the requirement of nucleotides, de novo pyrimidine synthesis gains prominence in rapidly proliferating cells [19]. Therefore, discerning whether CPT1A-mediated FAO as a carbon source promotes cellular nucleoside synthesis will provide clues to address the possible mechanism of cellular proliferation.

In this study, we addressed the issue of whether and how lipid metabolism affects cell cycle progression in NPC tumorigenesis mediated through CPT1A. Using a metabolomics approach, we illustrated a metabolic feature of active FAO in NPC cells. CPT1A participates in the regulation of intracellular neutral lipid content, which is one of the key molecules mediating abnormal activation of FAO in NPC cells. Our study establishes the oncogenic relevance of CPT1A and provides a mechanistic link from FAO to cell cycle regulation. The CPT1A-mediated FAO as a carbon source for pyrimidine synthesis and further confirmed that these metabolic adaptations provide intermediate metabolites and energy to support cell cycle-based proliferation.

## Results

### CPT1A is a key molecule for the abnormal activation of FAO in NPC

**Fig. 1:**
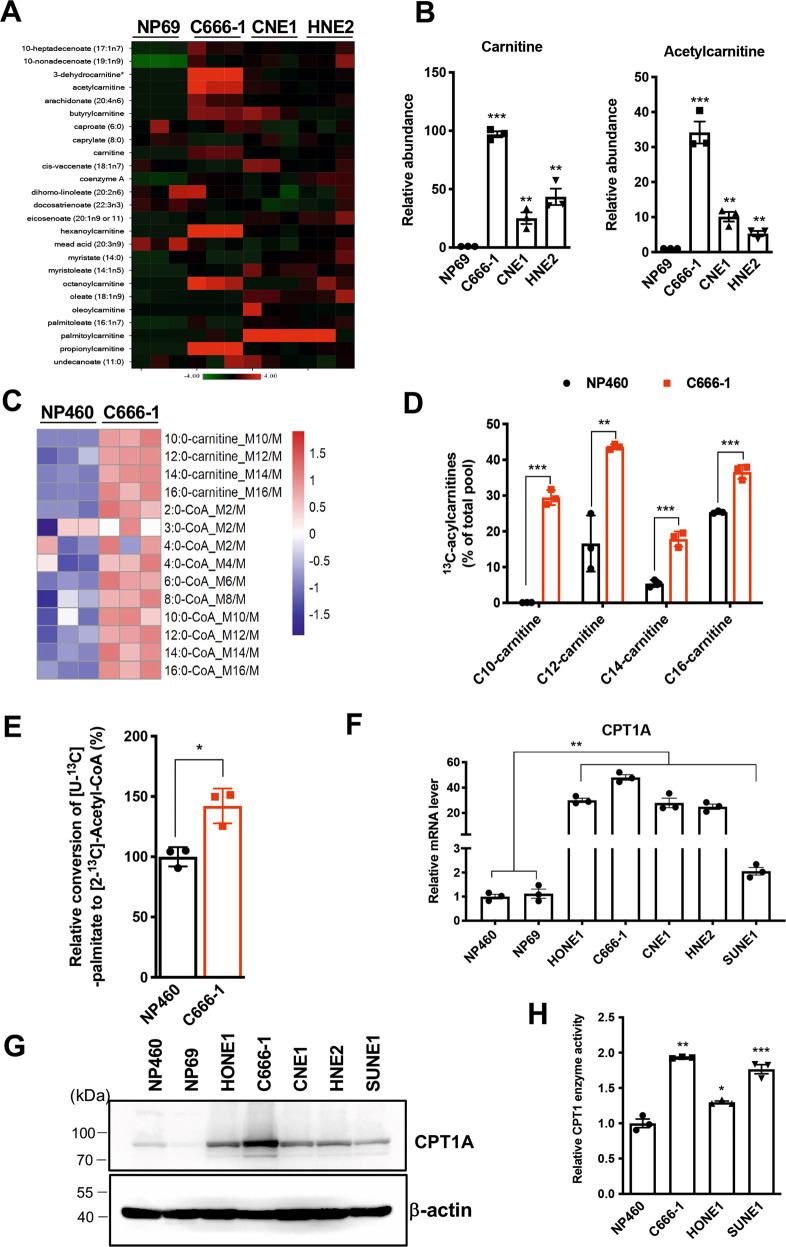
Abnormal activation of FAO is mediated by CPT1A in NPC cells. **A** Heatmap showing FAO of beta biochemicals in lysates from three replicates each of immortalized nasopharyngeal epithelial cells (NP69) and NPC cells (C666-1, CNE1, HNE2). **B** Fold changes in cellular carnitine and acetyl carnitine levels in NPC cells compared with immortalized nasopharyngeal epithelial cells (**P < 0.01, ***P < 0.001). **C** Heatmap of metabolic flux depicting ratios of detected FAO and carnitine shuttle pathway-related metabolites in NP460 and C666-1 cells. Significantly altered intermediate metabolites are indicated in blue text (decreased) and red text (increased). **D** Labeling incorporation from ^13^C_16_-palmitate into palmitoyl-carnitine (C16), myristoyl-carnitine (C14), lauroyl-carnitine (C12), and decanoyl-carnitine (C10) in C666-1 cells compared with NP460 immortalized nasopharyngeal cells. Data are shown as percentage of ^13^C_16_-C16, ^13^C_14_-C14, ^13^C_12_-C12, or ^13^C_10_-C10 compared to the total pool of each corresponding acylcarnitine. Three replicates were tested for each cell line (**P < 0.01, ***P < 0.001). **E** FAO activity was measured by [U-^13^C]-palmitate conversion to [2-^13^C]-acetyl-CoA in NP460 and C666-1 cells (*P < 0.05). **F** Real-time PCR was performed to examine CPT1A expression in the indicated cells (**P < 0.01). **G** Western blotting was performed to examine CPT1A expression in the indicated cells. **H** CPT1 enzymatic activity was measured in lysates of the indicated cells. Results are presented as fold changes and values represent mean ± SEM of three independent experiments (*P < 0.05, **P < 0.01, ***P < 0.001).

First, we used a metabolomics approach to construct global metabolic profiles, which revealed that overall metabolic levels are significantly different between immortalized nasopharyngeal epithelial cells and NPC cells. The specific metabolites of lipid metabolism were significantly different (Fig. 1A). Compared with NP69 cells, the level of carnitine and acetyl carnitine in C666-1 cells was much higher, as were those also in another two NPC cell types (Fig. 1B). This suggests that lipid metabolism might be in an active state in NPC cells. We further performed a targeted metabolomics analysis of acylcarnitine and acyl-CoA to confirm utilization of fatty acids through the carnitine shuttle system in NPC. Results indicated that acylcarnitine and acyl-CoA levels were significantly altered in C666-1 cells (Fig. 1C). Cells were incubated with uniformly labeled ^13^C16-palmitate; and the abundance of ^13^C-labeled palmitoyl-carnitine (C16), myristoyl-carnitine (C14), lauroyl-carnitine (C12), and decanoyl-carnitine (C10) were measured by ultra-high-performance liquid chromatography-tandem mass spectrometry (UPLC-MS/MS; Fig. 1D). All four acylcarnitines were considerably increased in C666-1 cells. Acetyl-CoA is a downstream metabolite of fatty acid carbon sources. Intracellular concentrations of acetyl-CoA were found to be increased in C666-1 cells (Fig. 1E), which suggests that intracellular palmitate was metabolized to acetyl-CoA. Thus we obtained more direct evidence for the abnormal activation of FAO in NPC cells.

To further investigate the mechanism of elevated FAO in NPC cells, we profiled the expression of a series of FAO-related genes by using a real-time PCR array (Supplementary Fig. S1A). Sequences of real-time PCR primers are listed in Table S1. We found that *CPT1A*, *acetyl CoA transferase (ACAA2)*, and *fatty acid transporters (FATP2)* are the most differentially expressed genes. We also examined the expression of three carnitine palmitoyl transferase isoenzymes *(CPT1A, CPT1B*, and *CPT1C)*, and determined that CPT1A is the only highly expressed carnitine palmitoyl transferase in NPC cells (Fig. 1F and Supplementary Fig. S1B). In addition, CPT1A protein expression levels and CPT1 enzyme activity are also increased (Fig. 1G, H).

### CPT1A promotes FAO, cell proliferation, and tumorigenesis of NPC

**Fig. 2:**
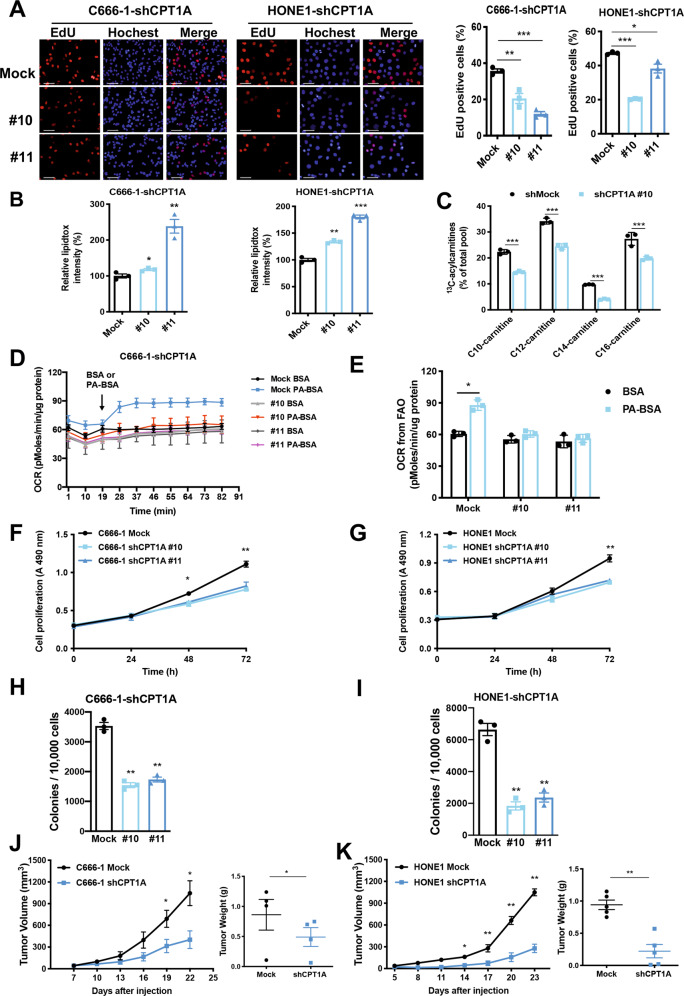
CPT1A promotes FAO, proliferation, and tumorigenic properties in NPC cells. **A** Cellular EdU incorporation was measured in C666-1 and HONE1 cells stably transfected with non-targeting control (GV248) or shCPT1A. The grouped graphs were analyzed as the ratio of the EdU-stained cells to the nucleus-stained cells (*P < 0.05, **P < 0.01, ***P < 0.001; scale bar = 50 μm). **B** CPT1A reduces intracellular neutral lipid content in NPC cells. Flow cytometry analysis of intracellular neutral lipid content by using the LipidTOX red probe in knockdown CPT1A NPC cells compared with control cells. Data represent the mean ± SEM of LipidTOX red intensity from three independent experiments (*P < 0.05, **P < 0.01, ***P < 0.001). **C** Labeling incorporation from ^13^C16-palmitate into palmitoyl-carnitine (C16), myristoyl-carnitine (C14), lauroyl-carnitine (C12), and decanoyl-carnitine (C10) in C666-1 cells stably transfected with *CPT1A* shRNA or control shRNA. Data are shown as percentage of ^13^C_16_-C16, ^13^C_14_-C14, ^13^C_12_-C12, or ^13^C_10_-C10 compared to the total pool of each corresponding acylcarnitine. Three replicates were tested for each cell line (***P < 0.001). **D** Oxygen consumption rates (OCR) were measured using the Seahorse XF-24 analyzer to evaluate FAO capacity. Arrows indicate the time when palmitate-BSA (175 μM) or BSA (33 μM) was added to CPT1A knockdown cells compared with control cells (*n* = 3). BSA was used as a control for palmitate. **E** The amount of OCR derived from FAO was quantified as response to PA-BSA substrate treatment. Data represent the mean ± SEM (*P < 0.05). **F**, **G** An MTS assay was performed to evaluate the effect of CPT1A on the proliferation of C666-1-shCPT1A (**F**) or HONE1-shCPT1A cells (**G**). Data represent the mean ± SEM from three independent experiments (*P < 0.05, **P < 0.01). **H**, **I** Colony-formation assay of C666-1-shCPT1A (**H**) or HONE1-shCPT1A cells (**I**). Data represent the mean ± SEM (**P < 0.01), *n* ≥ 3. **J**, **K** Left, the tumor growth curve of nude mice injected with knockdown CPT1A in C666-1 (**J**) or HONE1 cells (**K**). Data represent the mean ± SEM of tumor volume (mm^3^) for each group (C666-1 *n* = 4 per group; HONE1 *n* = 5 per group). Right, tumor weight of each group. Dots represent individual mice. Error bars represent mean ± SEM (*P < 0.05, **P < 0.01).

To clarify the effect of CPT1A on the proliferation of NPC cells, we generated C666-1-shCPT1A and HONE1-shCPT1A cells and SUNE1-CPT1A cells (Supplementary Fig. S1C–F). Sequences of shRNA to CPT1A are listed in Supplementary Table S2. Knockdown of CPT1A decreased the number of clones forming in both C666-1-shCPT1A and HONE1-shCPT1A cells. In contrast, overexpression of CPT1A increased colony formation in SUNE1-CPT1A cells (Supplementary Fig. S1G–I). Furthermore, we used the EdU labeling assay to detect the role of CPT1A in the proliferation of NPC cells. We found that knockdown CPT1A by two independent shRNAs decreased the proliferation of C666-1 and HONE1 cells (Fig. 2A).

By examining individual metabolic pathways and comparing the amount of multiple lipid metabolic metabolites, we realized that the overall lipid metabolism of NPC cells was substantially different from the immortalized nasopharyngeal epithelial cells (Supplementary Fig. S2A). When cellular FAO is activated, lipolytic catabolism increases, and neutral lipid content decreases. Changes in cellular fatty acid content were detected using the lipidTox red neutral stain, which indirectly reflects the level of lipid metabolism. Results showed that depletion of CPT1A significantly increased the fluorescence value of lipidTOX compared to control cells (Fig. 2B), suggesting that reduced CPT1A expression increased the neutral lipid content.

To further explore the utilization of fatty acids through the carnitine shuttle system, a ^13^C isotopomer tracing experiment was undertaken (Fig. 2C). All four acylcarnitines were considerably decreased in C666-1-shCPT1A cells. Palmitic acid is a basic fatty acid (16 carbon), which is a direct product of fatty acid synthesis and a direct substrate for the oxidation of fatty acids. The Seahorse cell metabolic dynamic analysis system was used to detect FAO status in CPT1A knockdown NPC cells. The oxidation efficiency of palmitic acid and the decrease in oxygen consumption (OCR) of C666-1-shCPT1A cells indicate a decrease in FAO activity (Fig. 2D, E). CPT1A was confirmed to be a key molecule mediating the abnormal activation of FAO in NPC cells.

Ethyl 2-[6-(4-chlorophenoxy) hexyl] oxirane-2-carboxylate (Etomoxir, Eto) is a homolog of palmitoyl-CoA, which can covalently irreversibly bind to the C-terminal active region of CPT1A and inhibit its enzymatic activity [20]. We observed an Eto dose-dependent growth inhibition of NPC cells (Supplementary Fig. S2B). Eto effectively inhibited ATP content in the immortalized nasopharyngeal epithelium NP460 and NPC cells by suppressing FAO (Supplementary Fig. S2C). Knockdown of CPT1A reduced ATP content in C666-1 cells (Supplementary Fig. S2D) and overexpression of CPT1A increased the ATP content of cells (Supplementary Fig. S2E). These results suggest that CPT1A, a key FAO enzyme, can promote energy metabolism in NPC cells.

Previous studies revealed that CPT1A is highly expressed in multiple cancers [21–24]. We performed the MTS assay to assess the effect of CPT1A expression on cell growth. Results (Fig. 2F, G) showed that knockdown of CPT1A decreased cell proliferation. While the percentage of PI-staining positive cells was not significantly affected (Supplementary Fig. S3A, B). Accordingly, CPT1A overexpression in SUNE1 cells conveys stronger growth advantages compared to control cells (Supplementary Fig. S3C). Furthermore, we utilized the soft agar colony-formation assay to test cellular anchorage-independent growth in vitro. The data show that knockdown of CPT1A significantly attenuated the cloning efficiency of NPC cells (Fig. 2H, I), and overexpression CPT1A augmented the cloning efficiency of SUNE1 cells (Supplementary Fig. S3D). Taken together, highly expressed CPT1A endows NPC cells with proliferative advantages.

In order to confirm the in vitro phenotypical changes induced by CPT1A, we implanted NPC cells into athymic nude mice, and growth of the xenografts was monitored accordingly. We found that knockdown of CPT1A significantly decreased tumor growth and tumor weight in vivo (Fig. 2J, K). In contrast, overexpression of CPT1A in SUNE1 cells increased the growth and tumor weight in vivo compared with the control group (Supplementary Fig. S3E). Thus, we demonstrated that highly expressed CPT1A has a potential role in promoting the proliferation and tumorigenicity of NPC cells.

### Genomic amplification of *CPT1A* in HNSCC

**Fig. 3:**
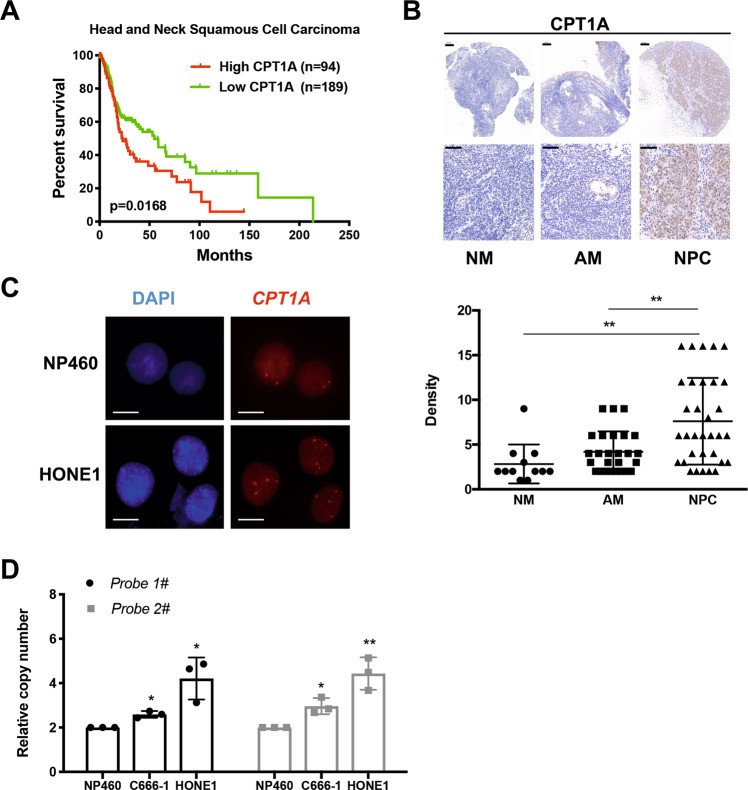
Genomic amplification of CPT1A in HNSCC. **A** Overall survival rates of head and neck squamous carcinoma patients with low (*n* = 189) or high (*n* = 94) *CPT1A* mRNA levels were estimated with the Kaplan–Meier method using the log-rank test and the TCGA database. **B** Representative IHC staining of CPT1A expression from a tissue microarray of normal nasal mucosa (NM), adjacent mucosa (AM), and nasopharyngeal carcinoma (NPC). (Scale bar, 100 μm; scale bar, 50 μm; **P < 0.01). **C** Analysis of *CPT1A* gene amplification by using FISH. Red signals are the target probe of the clone RP11-154D10, which covers the *CPT1A* gene (scale bar, 5 μm). **D** Copy number was determined in C666-1 and HONE1 cells by using the RT-qPCR method. NP460 were used as control cells and the *RPP14* gene was used as a reference gene. Two sets of primers (CPT1A probe 1# and CPT1A probe 2#) were used (*P < 0.05, **P < 0.01).

The *CPT1A* gene is located on chromosome 11q13.3, and this chromosomal region is reported to be amplified in NPC. In the TCGA dataset, 42 cases out of 279 head and neck tumors showed *CPT1A* gene amplification (Supplementary Fig. S4A). The copy number of *CPT1A* is also significantly correlated with its messenger RNA expression (*R* = 0.6872, *P* < 0.0001, unpaired *t* test; Supplementary Fig. S4B). Patients with high *CPT1A* mRNA levels had significantly lower overall survival (*P* = 0.0168), suggesting that CPT1A is closely associated with clinical outcome in HNSCC (Fig. 3A).

Using the NPC tissue chip (NPC961), which includes NPC samples and adjacent normal tissues, we found differences in the expression of CPT1A in nasopharyngeal mucosa, adjacent mucosa tissues, and NPC tissues (Fig. 3B). According to the IHC scores for CPT1A, we set the median score as the cut-off value and divided patients into two groups (Supplementary Fig. S4C). Among the nasopharyngeal carcinoma cases (*n* = 33) in the tissue chip, there were 14 patients with high CPT1A expression, and the percentage was 42.4%.

Here, we found that the expression of CPT1A was significantly stronger in NPC patients. In the TCGA database, *CPT1A* gene amplification was detected in 15% of patients with HNSCC. We performed fluorescence in situ hybridization (FISH) in NP460 and HONE1 cells by using probes for *CPT1A*. More than one hundred tumor cell nuclei from random areas were individually evaluated by counting red *CPT1A* signals (Fig. 3C). We further examined the copy number of the *CPT1A* gene in cell lines. Using the *RPP14* gene as an internal reference gene, the relative copy number of *CPT1A* was determined in C666-1 and HONE1 cells. The *CPT1A* gene copies in NPC cell lines were increased compared with the control NP460 cells (Fig. 3D). The increase in copy number may be one reason for the increased expression of CPT1A in NPC.

### Inhibition of FAO arrests cell cycle in the G1 phase

**Fig. 4:**
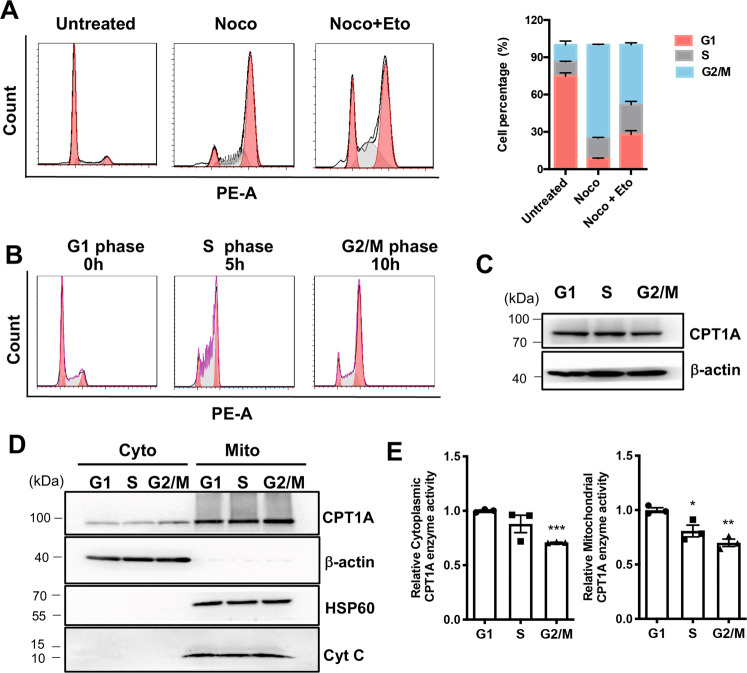
CPT1A has high enzymatic activity in the G1 phase of the cell cycle. **A** C666-1 cells after 6 h of 100 μM Eto treatment and the addition of 100 nM nocodazole after another 18 h. Cell cycle distribution was monitored by flow cytometry by using propidium iodide staining. Representative histogram data are shown. **B** C666-1 cells were synchronized into G1, S, and G2/M phases by using the HU double block, and cells were harvested for FACS analysis of cell cycle progression. **C** C666-1 cells were synchronized into G1, S, and G2/M phases by using the HU double block, followed by Western blotting for CPT1A expression. **D** C666-1 cells were synchronized at G1, S, and G2/M phases by using the HU double block, followed by western blotting of cytoplasmic and mitochondrial lysates for CPT1A expression. **E** C666-1 cells were synchronized at G1, S, and G2/M phases by using the HU double block, followed by measurement of CPT1A enzyme activity in cytoplasmic and mitochondrial lysates. Data represent the mean ± SEM (*P < 0.05, **P < 0.01, ***P < 0.001), *n* ≥ 3.

Eto is reported to inhibit the growth of tumor cells in prostate cancer, metastatic triple-negative breast cancer, and leukemia [21, 25, 26]. We added 100 μM Eto to cells and subsequently synchronized the cells with the microtubule inhibitor, nocodazole, which affects the cell cytoskeleton organization. Results showed that cells underwent G1 phase arrest by this manipulation (Fig. 4A). To analyze how cell cycle progression is coordinated in rapidly proliferating intracellular biosynthesis, we designed experiments to explore whether changes in FAO activity occur in different phases of the cell cycle. First, we performed cell cycle synchronization of C666-1 cells using the hydroxyurea (HU) double blocking method, collected cells at designated time points, and measured the cell cycle distribution by flow cytometry (Fig. 4B). CPT1 is incorporated into the mitochondrial outer membrane by the insertion of two transmembrane domains, with its N-terminus and C-terminus facing the cytoplasm [27]. We further investigated the expression level of the CPT1A protein by western blotting and results indicated that no significant change was observed in cytoplasmic or mitochondrial component proteins (Fig. 4C, D). Interestingly, CPT1A enzyme activity reached its highest value in the G1 phase both in cytoplasmic and mitochondrial fractions (Fig. 4E).

To confirm the role of CPT1A in the regulation of G1-cell cycle-related molecules, we treated HONE1 cells with 100 μM Eto for 24 h. Results showed that compared with the control group, Eto could induce increased phosphorylation of AMPK (Thr172) and GSK3β (Ser9), downregulate expression of cyclin D1 and CDK4, and phosphorylation of Rb (Thr821), but did not affect the levels of total Rb (Supplementary Fig. S4C). Proteasome inhibitor MG132 treatment reversed Eto-induced downregulation of cyclin D1 expression in C666-1 cells (Supplementary Fig. S4D). This suggests that Eto may induce G1-cell cycle arrest in NPC cells through proteasome degradation of cyclin D1.

### Fatty acids as a carbon source for cell cycle progression

**Fig. 5:**
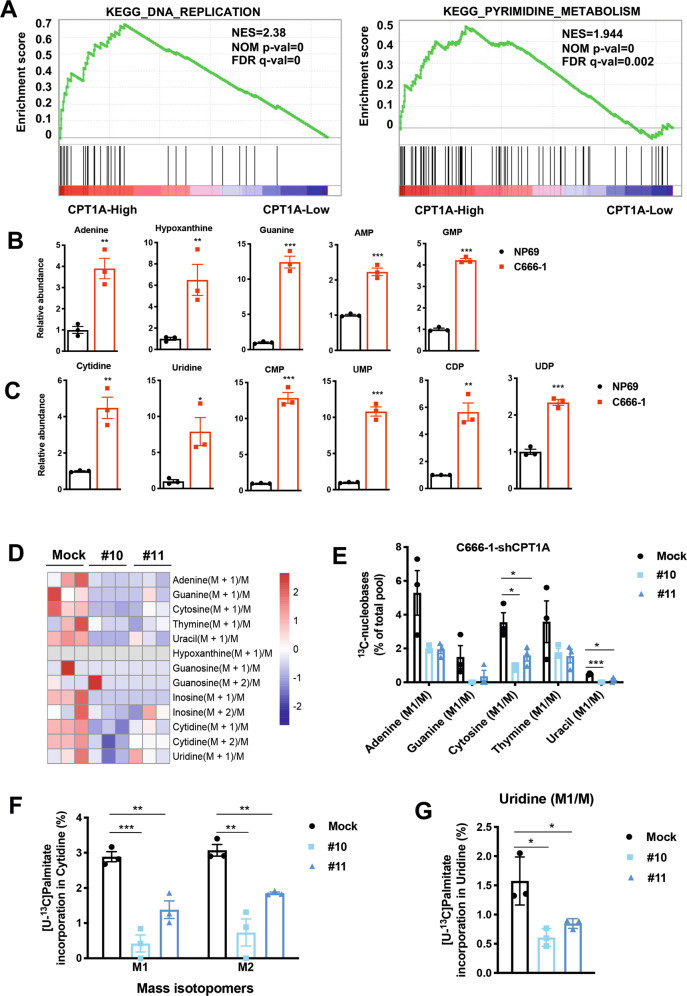
Increased abundance of nucleotide metabolic intermediates through CPT1A-mediated FAO in NPC cells. **A** GSEA plot correlating *CPT1A* mRNA levels with KEGG DNA replication and the pyrimidine metabolic pathway based on publicly available NPC patient gene-expression profiles (NCBI/GEO/GSE12452, *n* = 41). **B** Box diagram with fold changes in cellular purine metabolite levels in C666-1 cells compared with NP69 cells. Three replicates were tested for each cell line (**P < 0.01, ***P < 0.001). **C** Box diagram with fold change of cellular pyrimidine metabolite levels in C666-1 cells compared with NP69 cells. Three replicates were tested for each cell line (*P < 0.05, **P < 0.01, ***P < 0.001). **D** Heatmap of metabolic flux depicting ratios of detected purine and pyrimidine nucleoside pathway-related metabolites in C666-1 knockdown CPT1A cell lines. Significantly altered intermediate metabolites are indicated in blue text (decreased) and red text (increased). **E** Labeling incorporation from ^13^C-palmitate into nucleobases in C666-1 cells stably transfected with *CPT1A* shRNA or control shRNA. Data are shown as percentage of ^13^C-adenine, ^13^C-guanine, ^13^C-cytosine, ^13^C-thymine, or ^13^C-uracil compared to the total pool of each corresponding nucleobase (*P < 0.05, ***P < 0.001). **F** Percentage M + 1 and M + 2 labeling from [U-^13^C] palmitate in cytidine in C666-1 cells stably transfected with *CPT1A* shRNA or control shRNA (**P < 0.01, ***P < 0.001). **G** Percentage M + 1 labeling from [U-^13^C] palmitate in uridine in C666-1 cells stably transfected with *CPT1A* shRNA or control shRNA (*P < 0.05).

We analyzed the expression of *CPT1A* and other genes that are regulated by various signaling pathways by using gene set enrichment analysis (GSEA) in the publicly available NPC patients’ expression profiles (GSE12452). We found that CPT1A expression levels in NPC showed a positive correlation with the DNA replication and pyrimidine metabolic pathway (Fig. 5A). Further, we analyzed the nucleic acid-related metabolites and found that higher levels of purine (Fig. 5B) and pyrimidine (Fig. 5C) metabolites in C666-1 cells compared with NP69 cells.

In endothelial cells, fatty acid intermediates provide building blocks for biosynthesis pathways such as dNTP synthesis [18]. Therefore, we determined whether intermediate metabolites and products in the biosynthesis pathways were changed accordingly by tracing ^13^C-labeled palmitate (Fig. 5D and Supplementary Fig. S4F). We observed ^13^C incorporation of thymine, uracil, and uridine in C666-1 cells at ~2–4%, but not in NP460 cells. Consistently, the proportions of ^13^C incorporated adenine, guanine, cytosine, thymine, and uracil were decreased in CPT1A knockdown cells. Importantly, CPT1A inhibition by shRNA significantly lowered the fraction of ^13^C-palmitate-derived carbons into nucleosides (Fig. 5E).

As a whole, these results showed that [U-^13^C] palmitate is a carbon fuel for pyrimidine in NPC cells acting mainly through its shuttle and oxidation to acetyl-CoA to promote nucleoside synthesis. We therefore assessed whether impaired nucleoside synthesis caused the proliferation defect of interfering CPT1A in NPC cells. Indeed, labeled carbons from [U-^13^C] palmitate were incorporated into cytosine and uridine, and this incorporation was reduced with CPT1A silencing (Fig. 5F, G). The increased content of nucleoside metabolic intermediates in NPC cells promotes the biological functions of the abnormally high expression of CPT1A at the point of metabolic remodeling involved in cell cycle regulation.

### Targeting periodic activation of CPT1A-mediated FAO hinders the cell cycle G1/S transition

**Fig. 6:**
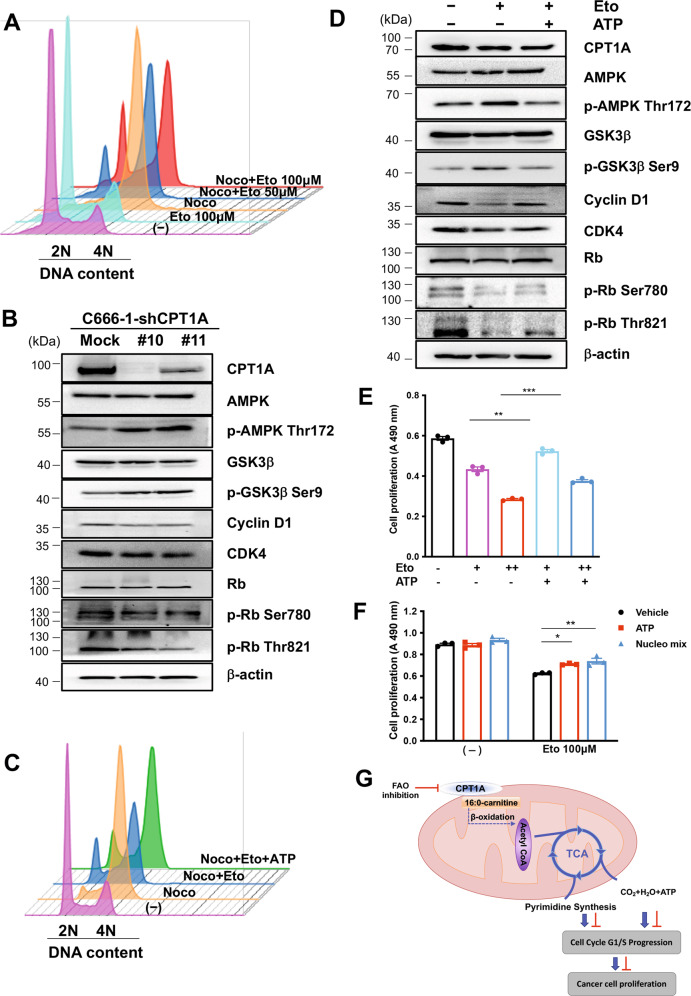
CPT1A is involved in modulating G1 phase cell cycle regulatory proteins. **A** Cell cycle distribution after release from G2/M synchronization with a G1 population of C666-1 cells treated with different concentrations of Eto. **B** Western blot analysis of the effect of *CPT1A*-targeting shRNAs and the expression of G1 phase markers (cyclin D1, CDK4, Rb) in C666-1 cells. β-Actin was used as a control to confirm equal loading of protein. **C** Cell cycle distribution after release from G2/M synchronization with a G1 population of C666-1 cells treated with Eto and ATP (50 μM). **D** Western blotting to detect cell cycle-related molecules (cyclin D1, CDK4, Rb) in C666-1 cells treated or not treated 24 h with Eto (100 μM) and ATP (50 μM). β-Actin was used as a control to confirm equal loading of protein. **E** An MTS assay showing proliferation of C666-1 cells treated with different concentrations of Eto (100 μM or 200 μM) and ATP (50 μM). Results are plotted as the mean absorbance at 490 nm ±SEM of three independent experiments (**P < 0.01, ***P < 0.001). **F** An MTS assay showing proliferation of C666-1 cells treated with Eto and ATP (50 μM) or a nucleo mix (25 μM). Results are plotted as the mean absorbance at 490 nm ±SEM of three independent experiments (*P < 0.05, **P < 0.01). **G** Schematic illustrating that CPT1A mediates FAO to maintain nucleotide metabolic intermediates and ATP levels to promote cell cycle regulation and excessive cell proliferation.

We treated cells with different concentrations of Eto, and the superimposed cell cycle flow results showed that inhibition of FAO blocked synchronized cell cycle progression in the G1 phase (Fig. 6A). Detection of G1 phase regulatory molecular markers in the cell cycle revealed that knockdown of CPT1A increased phosphorylation of AMPK (Thr172) and GSK3β (Ser9), reduced the expression levels of cyclin D1 and CDK4, and decreased Rb (Thr821) phosphorylation (Fig. 6B). These results indicate that inhibition of FAO decreased expression of cell cycle G1-related proteins and interfered with downstream Rb signaling pathways.

To further verify that Eto treatment caused G1 arrest by energy loss or downregulation of nucleic acid metabolites, we added ATP to reverse Eto-induced G1 arrest (Fig. 6C). Treatment with Eto significantly increased the phosphorylation levels of AMPK (Thr172) and GSK3β (Ser9). Consistently, the addition of ATP could reverse phosphorylation of AMPK and GSK3β, but the expression of cyclin D1 and CDK4 and the phosphorylation level of Rb (Ser780, Thr821) increased in extra ATP-treated cells (Fig. 6D). However, we observed that the addition of ATP or a mixture of nucleosides partially reversed cell proliferation inhibition (Fig. 6E, F), and no significant effect on cell morphology and death (Supplementary Fig. S5A, B). Supplementation with a mix of nucleosides or ATP also partly rescued the G1 phase arrest caused by Eto treatment in C666-1 cells, indicating that FAO blockade impaired nucleoside synthesis. Based on these results, CPT1A mediated FAO to maintain nucleoside metabolism intermediate levels, which are critical for NPC cell cycle progression and proliferation (Fig. 6G).

## Discussion

Dysregulated lipid metabolism represents an important metabolic alteration in cancers. We illustrated the metabolic features of FAO in NPC cells, and identified CPT1A as a key molecule for the abnormal activation of FAO.

Multiple cancers show increased glucose uptake and enhanced glycolytic rates [28, 29], which supports the production of intermediates for the synthesis of lipids, proteins, and nucleic acids [30]. Also, cancer cells have increased glutamine uptake and glutaminolysis, which replenish intermediates of the tricarboxylic acid cycle [31]. Indeed, to sustain high proliferation rates, cancer cells may use a wide variety of substrates and substrate sources to meet their catabolic and anabolic needs, including internally- and externally derived fatty acids [32]. Deregulation of fatty acid metabolism can contribute to tumorigenesis by regulating proliferation, apoptosis, migration, and tissue invasion of transformed cells [33, 34]. Previous data have also linked CPT1A to tumor progression [35–37]. Enzymes involved in FAO, such as CPT1, might be considered a potential target in oropharyngeal squamous cell carcinomas (OPSCC) [6]. In this study, we provide evidences showing that CPT1A is a key metabolic enzyme of FAO in NPC, driving the rapid proliferation links for cellular metabolism and cell cycle progression. Previous studies indicated that AMPK phosphorylation activation, and downregulate cyclin D1 protein levels [38–40]. Also, cyclin D is a highly unstable protein, and its ubiquitination and degradation occur in late G1 and S phases. Cyclin D1 is a metabolic checkpoint and a regulator of cell cycle machinery in cancer cells. Studies have determined that Thr286 phosphorylation-dependent degradation of cyclin D1 is regulated by kinases such as ERK2, p38, and GSK3β [41–43]. Cyclin D1 degradation has been reported to occur through the AMPK/GSK3β signaling axis through the proteasome pathway in ovarian cancer cells [44]. In our work, with decreased cellular ATP levels, AMPK is activated as a master metabolic switch to maintain energy homeostasis. Changes in the signaling axis of CPT1A/AMPK/GSK3β/cyclin D1 were observed, suggesting that CPT1A-mediated FAO promotes the activation of the signaling axis during the G1 phase. CPT1A links FAO to cell cycle progression and promotes cell proliferation and growth. Targeting CPT1A-mediated-FAO hinders the cell cycle G1/S transition.

Increased genomic disruption has been associated with poor prognosis in many cancer types [45]. Metabolic gene amplification accompanies canonical oncogene drivers in cancer [46]. Amplification of the 11q13 chromosome region has been reported in advanced OPSCC [47], HNSCC [48, 49], and esophageal carcinoma [13]. This often results in those gene overexpression and is accompanied by worse survival rates and aggressive phenotypes. High expression of CPT1A in head and neck tumors is associated with reduced overall survival of patients. Here, we found that CPT1A amplification is likely to be an important driver in NPC progression. An increased level of CPT1A mediates lipid metabolic pathways to generate ATP and promotes the malignant phenotypes of NPC cells.

Nucleotides are basic molecules needed for DNA synthesis, in which glutamine provides the nitrogen that is required for purine and pyrimidine de novo nucleotide synthesis [50]. Endothelial cells metabolize fatty acids to acetyl-CoA to sustain the tricarboxylic acid cycle, in conjunction with an anaplastic substrate, to facilitate dNTP synthesis for EC proliferation [51]. CPT1A can catalyze the rate-limiting step of the FAO pathway, which may represent an alternate carbon source for anabolic processes of dNTP [18]. Targeting CPT1A has shown positive results for impairing cancer cell survival and inhibiting tumor cell proliferation in vitro and in vivo models of Burkitt’s lymphoma [25]. Our results suggest that acetyl-CoA production fueled by mitochondrial FAO provides the cells with an additional mechanism for producing the quantity of intermediates of pyrimidine metabolism to facilitate the demands of cell cycle progression. Labeled carbons from [U-^13^C] palmitate were incorporated into the pyrimidine precursors, cytidine and uridine, and this incorporation was reduced upon CPT1A silencing. Indeed, ATP and nucleotide supplementation restored the proliferation rates to control levels in Eto-treated cells. CPT1A mediates FAO to promote tumor cell proliferation and identify fatty acids as providing a carbon source for the synthesis of nucleoside metabolic intermediates by metabolic flux analysis. Knockdown of CPT1A significantly lowered the fraction of ^13^C-palmitate-derived carbon into pyrimidines. Consequently, the knockdown of CPT1A attenuated fatty acid utilization and eliminated the pro-proliferation advantage provided by pyrimidine synthesis in NPC.

In summary, this study elucidates the metabolic profile of FAO in NPC and identifies CPT1A as a key molecule in the aberrant activation of FAO. Our findings provide evidence for a novel function of CPT1A-mediated FAO to maintain nucleoside metabolic intermediate levels, which support cellular proliferation, survival, and metabolic adaptation in NPC. This molecular mechanism is based on the link between energy production and tumor cell growth.

## Materials and methods

### Cell lines and culture conditions

The immortalized NP69 and NP460 nasopharyngeal epithelial cell lines were cultured in keratinocyte serum-free medium (Invitrogen, Carlsbad, CA, USA). The NPC cell lines, including CNE1, CNE2, C666-1, C666-1-shCPT1A, HK1, HONE1, HONE1-shCPT1A, HNE1, HNE2, HNE3, SUNE1, and SUNE1-CPTA were cultured in RPMI-1640 medium (Hyclone, Logan, UT, USA) supplemented with 10% FBS (Hyclone). All cells were cultured at 37 °C in a humidified incubator with 5% CO_2_. Cells were cytogenetically tested and authenticated before being frozen. All cell lines were regularly tested for mycoplasma contamination by using a PCR-based assay (#A8994; AppliChem).

### Metabolic profiling

Metabolomic profiles were obtained to assess the relative distribution of various cellular metabolites of immortalized nasopharyngeal epithelial and NPC cells. Cells were collected and quickly frozen. Further sample preparation, metabolic profiling, peak identification, and curation were performed using Metabolon (Durham, NC, USA) following their described methods.

### ^13^C tracer experiments and metabolite levels

For metabolic flux experiments, ^13^C-palmitate tracing was conducted using LC/MS at LipidALL Technologies. Cells (1 × 10^7^) were incubated for 8 h at 37 °C in RPMI-1640 medium with 10% FBS. For ^13^C-palmitate incorporation in metabolites, cells were incubated in growth medium with 50 μM [U-^13^C] palmitate for 24 h. Then, cells were quickly washed with 1×PBS and fixed with pre-cooled methanol (HPLC-MS grade, Millipore) for 30 min at −80 °C. The extraction method was referenced to a previous paper, but with modification [52]. Samples were incubated for 30 min at 1500 rpm and 4 °C and then centrifuged for 10 min at 12,000 rpm and 4 °C. The supernatant fractions were placed into clean 1.5-ml centrifuge tubes and dried using a SpeedVac. The dried extracts were dissolved with 50% acetonitrile in water and the upper layer liquids were collected for LC–MS analysis. The InfinityLab Poroshell 120 HILIC-Z column (2.1 mm × 50 mm, 2.7 μm, Agilent Technologies, Germany) was used in this study. Ultra-performance Liquid Chromatography (Agilent 1290 II, Agilent Technologies, Germany) coupled to the Quadrupole-TOF MS 5600 Triple TOF Plus, AB SCIEX, Singapore) was used to acquire metabolome data.

### Metabolic flux analysis of FAO

FAO activity was measured by monitoring the conversion rate of [U-^13^C]- palmitate to [2-^13^C]-acetyl-CoA by using LC/MS. In brief, the cells were incubated with 50 μM [U-^13^C]-palmitate conjugate for 24 h. After incubation, the metabolites were extracted and dried and then derivatized with MSTFA. The FAO activity was calculated by dividing the acetyl-CoA labeling rate by the palmitate uptake rate.

### Acyl-CoA and acylcarnitine quantification by LC/MS

Acyl-CoAs and acylcarnitines were extracted as previously described [53]. Briefly, 300 µL of extraction buffer containing isopropanol, 50 mM KH_2_PO_4_, and 50 mg/mL BSA (25:25:1 v/v/v) acidified with glacial acetic acid were added to cells. Next, lipids were extracted by incubation at 4 °C for 1 h and centrifuged at 1500 rpm. Then 300 µL of petroleum ether were added and the sample was centrifuged at 12,000 rpm for 2 min at 4 °C. The upper phase was removed. The samples were extracted two more times with petroleum ether as described above. To the lower phase finally remaining, 5 µL of saturated ammonium sulfate were added followed by 600 µL of chloroform: methanol (1:2 v/v). The sample was then incubated on a thermomixer at 450 rpm for 20 min at 25 °C, followed by centrifugation at 12,000 rpm for 5 min at 4 °C. The clean supernatant fraction was transferred to a fresh tube and subsequently dried in a SpeedVac under the OH mode (Genevac). Dried extracts were resuspended in an appropriate volume of methanol: water (9:1 v/v) prior to liquid chromatography–mass spectrometry (LC–MS) analyses on a Thermofisher U3000 DGLC coupled to the Sciex QTRAP 6500 Plus. Levels of individual acyl-CoAs and acylcarnitines are expressed as extracted intensities.

### Immunohistochemistry analysis of NPC tissue array

The NPC tissue array was purchased from Pantomics (Richmond, CA, USA) and follow-up data and clinical details were provided by this company and are available online. Immunohistochemistry was performed as previously described [54]. Images of the sections were acquired and differentially quantified by pathologists. An NPC tissue chip (NPC961) included 36 cases of NPC, 31 cases of adjacent mucosa (AM), and 12 cases of the normal nasal mucosa (NM). However, 33 cases of NPC, 26 cases of AM, and 12 cases of NM were actually available for analysis. Primary CPT1A (Abcam, USA) antibodies were diluted in serum blocking solution and were incubated overnight at 4 °C. HRP-labeled rabbit or mouse secondary antibodies were applied respectively for 1 h. ICC was developed using the DAB Chromogen System (Dako, USA) and the nuclei counterstained with hematoxylin.

### EdU labeling and staining

The EdU labeling and staining in NPC cell lines were performed with a BeyoClick EdU cell proliferation kit with Alexa Fluor 647 (C0081L, Beyotime) according to the manufacturer’s instructions. The high-content cell imaging system (Operetta, Perkin Elmer) was used for analysis and photography.

### Anchorage-independent growth

For EGF-induced transformation, cells (8 × 10^3^/mL/well) were exposed to EGF in 1 mL of 0.3% Basal Medium Eagle agar containing 10% FBS. Cultures were maintained in a 37 °C, 5% CO_2_ incubator. For anchorage-independent growth, cells were seeded into six-well plates with 0.3% Basal Medium Eagle agar containing 10 or 20% (C666-1) FBS and cultured. Colonies were scored using a microscope and Image-Pro PLUS (v.6) software program (Media Cybernetics).

### Tumorigenicity assay

Tumorigenicity assays were maintained and manipulated according to guidelines established by the Medical Research Animal Ethics Committee of Central South University. The six-week-old female athymic nude mice (BALB/C) were purchased from SLAC Laboratory Animal Co. Ltd. (Changsha, China). The nude mice were randomly grouped. Then 5 × 10^6^ cells per animal were injected into the subcutaneous tissue over the right flank regions of nude mice. The tumors were measured every 3 days. Tumor volume was calculated using the following formula: V = a × b^2^ /2, where “a” and “b” were the shortest and the longest diameters, respectively. At the end of the experiments, mice were euthanized by CO_2_ inhalation, and the weight of extracted xenograft tumors was obtained at the same time.

### Western blot analysis

After electrophoretic separation and immunoblotting of whole-cell lysates, blots were incubated with appropriate primary antibodies and secondary antibodies labeled with horseradish peroxidase. After blocking, the blots were incubated overnight at 4 °C with different antibodies against β-actin (Sigma, USA), cyclin D1, CDK4, Rb, AMPK, GSK3β (Cell Signaling Technology, MA, USA), E2F (Santa Cruz, CA, USA), or CPT1A (Abcam, USA) followed by incubation on the following day at room temperature with the secondary rabbit or mouse antibody (Cell Signaling Technology, USA). Antibodies are listed in Supplementary Table S3. The ECL Detection Kit (Engreen Biosystem, China) was used to develop the blots. Visualization of proteins was performed using the ChemiDoc XRS system with Image Lab software.

### Cell fractionation

Cells were scratched from the dish and at least 2 × 10^7^ cells were collected and then washed twice with ice-cold PBS. Then, the cells were resuspended in 1 ml MSHE + BSA buffer, gently mixed, and placed on ice for 5 min. Full grinding was slowly performed about 60 times at 4 °C for 10 min. The cells were centrifuged for 30 min at 12,000×*g* at 4 °C. The supernatant fractions were collected, and the cytosol CPT1A level was analyzed by Western blotting. Then, MSHE + BSA buffer (200 μL) was added to the cell pellet, and the mixture was mixed and centrifuged at 13,000 rpm for 10 min at 4 °C. The pellet was resuspended in 80 μL of IP lysis buffer and left on ice for 30 min to obtain the mitochondrial proteins, and mitochondrial CPT1A was analyzed by western blotting.

### ATPLite assay to detect the content of ATP in NPC cells

The ATP in the cell can react with fluorescein and generate fluorescence under the effect of luciferase. The kit used was the ATPLite Luminescence Assay System (Waltham, MA, USA). Cells were seeded in a 96-well plate at a density of 8 × 10^3^/well in triplicate and after incubation for 6 h, cells were then treated or not treated with Eto for 24 h. Cell lysis solution (50 μL) was added and then the plate was mixed by shaking for 5 min. Then substrate solution (50 μL) was added and the plate continued to mix by shaking at a speed of 700 rpm in the dark for an additional 10 min. Fluorescence intensity was detected and the ATP content was calculated.

### Seahorse XF-24 cell metabolic dynamic analyzer to detect FAO

The Seahorse XF cell metabolic dynamic analyzer (Seahorse Bioscience) can monitor real-time cellular oxygen consumption, extracellular acidification rate, and the basic index of the mitochondrial respiratory function and reaction. Adding palmitic acid to cells after starvation treatment activates oxidation and reflects the level of FAO in the cells by detecting changes in oxidative respiration of mitochondria. The day before the experiment, the cells were placed into XF MPP and the hydrate casing package was stored at 37 °C stores overnight. At the beginning of the second day of the experiment, the number of cells and cell density were ensured to be at least 90% with cells in monolayers evenly distributed. At the 19th minute, palmitate (175 μM) was added and the oxygen consumption rate of C666-1 cells was detected. The data were then analyzed after normalization.

### FISH assay

Interphase FISH analysis was performed according to the manufacturer’s instructions (www.abbottmolecular.com). Fluorescent images were captured and analyzed by using a Leica DM500B fluorescence microscope and Leica CW400 image analysis software. The bacterial artificial chromosome (BAC) clone probe specific for the *CPT1A* gene (RP11-154D10, red) was applied.

### Carnitine palmitoyl transferase-1 enzymatic activity

Carnitine palmitoyl transferase-1 enzymatic activity was measured as previously described [7]. Cells were harvested after cell cycle synchronization as whole-cell lysates and mitochondrial/cytoplasmic proteins were prepared. The DTNB buffer and cell lysate were mixed and incubated at room temperature for 30 min. The absorbance of buffer alone at 405 nm was defined as background. Then 100 μM palmitoyl-CoA and 5 mM l-carnitine were added to the mixture. After a 20 min incubation at 37 °C, the absorbance was measured at 405 nm. The difference between readings with and without substrates was normalized to the protein concentration. CPT1 enzymatic activity is expressed as millimoles of CoA-SH released per milligram of protein.

### Cell cycle synchronization

Cellular microtubule polymerization is inhibited by the mitotic inhibitor, nocodazole. Cell growth was synchronized to the G2/M phase and the percentage of nocodazole treated (18 h) G2/M phase cells was significantly higher compared to the control group.

The hydroxyurea (HU) blocking method was used to synchronize C666-1 cells in the G1/S phase. Hydroxyurea (2.5 mM) was added to the complete medium and cells incubated for 23 h. Cells were then rinsed with fresh medium twice and new complete medium was then added and incubated for 10 h. Hydroxyurea (2.5 mM) was then added and cells incubated for 13.5 h a second time. G1/S phase cells were harvested by trypsin digestion and the synchronized cells were divided into two new dishes. The S phase or G2/M phase cells were collected after culturing for 5 or 10 h, respectively.

### Quantification and statistical analysis

All statistical calculations were performed with the GraphPad Prism 6 software program (GraphPad Software). The experimental data are presented as the mean value ± SEM. The statistical significance of the data was analyzed using ANOVA or a standard Student’s *t* test. Overall and disease-free survival were determined by the Kaplan–Meier method and compared using a log-rank test. A *P* value of < 0.05 was considered statistically significant.

## Supplementary information

Supplementary Material

Original Blot 1

Original Blot 2

Original Blot 3

Original Blot 4

Original Blot 5

Reproducibility checklist

## Data Availability

The TCGA data referenced during the study are available in a public repository from the cBioPortal for Cancer Genomics, TCGA website (http:// www.cbioportal.org/). Oncoprint (cBioPortal) for the HNSCC TCGA provisional cohort of tumors with complete data (sequencing, copy number alterations, and mRNA expression) showing tumors with percentages are relative to the complete number of tumors in the cohort (*n* = 279).
