# Supplementary material for: CPT1A-mediated fatty acid oxidation promotes cell proliferation via nucleoside metabolism in nasopharyngeal carcinoma
Source: Cell Death Dis. 2022 Apr 11;13(4):331. doi: 10.1038/s41419-022-04730-y (PMC9001659; doi:10.1038/s41419-022-04730-y)
Supplement: Supplementary file 1 — Supplementary Material [file 41419_2022_4730_MOESM1_ESM.docx]

**Supplementary Materials**

**Table S1.** **Sequences of primers for quantitative real-time PCR analysis**

| Gene Name | Sequences of primers |
| --- | --- |
| *β-actin* | F: 5′- CCTTGCACATGCCGGAG-3′ |
|  | R: 5′-GCACAGAGCCTCGCCTT-3′ |
| *CACT* | F: 5′- TGTGGTGAATACGCCAGATAAC-3′ |
|  | R: 5′-ACAGGGGTTCAGACTGCTATT -3′ |
| *CPT1A* | F: 5′-ATCAATCGGACTCTGGAAACGG-3′ |
|  | R: 5′-TCAGGGAGTAGCGCATGGT-3′ |
| *CPT2* | F: 5′-CATACAAGCTACATTTCGGGACC -3′ |
|  | R: 5′-AGCCCGGAGTGTCTTCAGAA -3′ |
| *ACADM* | F: 5′-ACAGGGGTTCAGACTGCTATT -3′ |
|  | R: 5′- TCCTCCGTTGGTTATCCACAT-3′ |
| *ACADS* | F: 5′-AGGGCGACTCATGGGTTCT -3′ |
|  | R: 5′-GGGATGCGACAGTCCTCAAAG -3′ |
| *ACADVL* | F: 5′-TCAGAGCATCGGTTTCAAAGG -3′ |
|  | R: 5′-AGGGCTCGGTTAGACAGAAAG -3′ |
| *ACAD9* | F: 5′-CTCAAGACTAGGGGAGATCATCA -3′ |
|  | R:5′-ACGCCAGTTTAGGCAAGTATTT -3′ |
| *ACAD10* | F: 5′-CGAGTGGCAAAGCAGTTCC -3′ |
|  | R: 5′-CCATGCAGGACTCCACAATCA -3′ |
| *ACAD11* | F: 5′-TTGGATTCCCCGTTCCCAAG -3′ |
|  | R: 5′-AAATCACGGAAGATTCGACCC -3′ |
| *ACAA2* | F: 5′-AGACAATGCAGGTAGACGAGC -3′ |
|  | R: 5′-ACCCATGATAGAGGGATCACATC -3′ |
| *ECI1* | F: 5′-CTGCGGTTGTACCAGTCCAA -3′ |
|  | R: 5′-TCCTGGTATCGAGTGATGATGT -3′ |
| *ECI2* | F: 5′- ATGGGACGCATGGAATGCC-3′ |
|  | R: 5′-TTCAAACCCAGTTGATTTCCTGT -3′ |
| *FATP2* | F: 5′-GGCGCTCCTTATGGGTAACG -3′ |
|  | R: 5′-CTTGGCAGTATCTCTTCGACAG -3′ |
| *FATP4* | F: 5′-GGACCCAGGTGGGATTCTC -3′ |
|  | R: 5′-CGCGCCTGATGGTCTTGAT -3′ |
| *VLDLR* | F: 5′-CTGTGTAAAGAAGACGTGTGCT -3′ |
|  | R: 5′-TGATTTCATGTATGCGGCATGT -3′ |
| *CPT1B* | F: 5′-CCTGCTACATGGCAACTGCTA -3′ |
|  | R: 5′-AGAGGTGCCCAATGATGGGA -3′ |
| *CPT1C* | F: 5′-GGGCCGCTTTCTTTGTGTC -3′ |
|  | R: 5′- AGAAGACGATTAGGGTGAAGGAT-3′ |

**Table S2. Sequences used to construct shRNA of CPT1A**

| **shCPT1A** | **Sequences** |
| --- | --- |
| 7# | CGTGTAAATGTGGAAAGCCTT |
| 8# | CGACCGGTTTAGGTGATGTTA |
| 9# | CCAAGGATGTACATGACAACG |
| 10# | GCCCTCTTATAAGAAGAGACA |
| 11# | TTCGTTAGATGCGACGTGTTC |

**Table S3. Antibodies and chemicals list**

| **Reagent or Resource** | **Source** | **Identifier** |
| --- | --- | --- |
| **Antibody** | | |
| CPT1A | Abcam | Cat# ab128568 |
| CDK4 | CST | Cat# 12790 |
| Cyclin D1 | CST | Cat# 2978 |
| HSP60 | Santa | Cat# SC-13115 |
| RB | CST | Cat# 9309 |
| p-RB Ser780 | CST | Cat# 9307 |
| p-RB Thr821 | Invitrogen | Cat# 44582G |
| AMPK | CST | Cat# 2532S |
| p-AMPK Thr172 | CST | Cat# 2531S |
| GSK3β | CST | Cat# 9315 |
| p-GSK3β Ser9 | CST | Cat# 9323 |
| Cytochrome c | CST | Cat# 4272S |
| V5 | Invitrogen | Cat# R96025 |
| β-actin | Sigma-Aldrich | Cat# A5441 |
| **Chemicals** | | |
| LipidTOX | Invitrogen | Cat# H34476 |
| ^13^C_16_-palmitate | Sigma-Aldrich | Cat# 605573 |
| Etomoxir | Sigma-Aldrich | Cat# E1905 |
| ATP | Sigma-Aldrich | Cat# 10519979001 |
| Nucleo Mix | Sigma-Aldrich | Cat# ES-008-D |
| Hydroxyurea | Sigma-Aldrich | Cat# H8627 |
| Nocodazole | Sigma-Aldrich | Cat# 31430-18-9 |
| MG-132 | MCE | Cat# HY13259 |

**Figure S1**


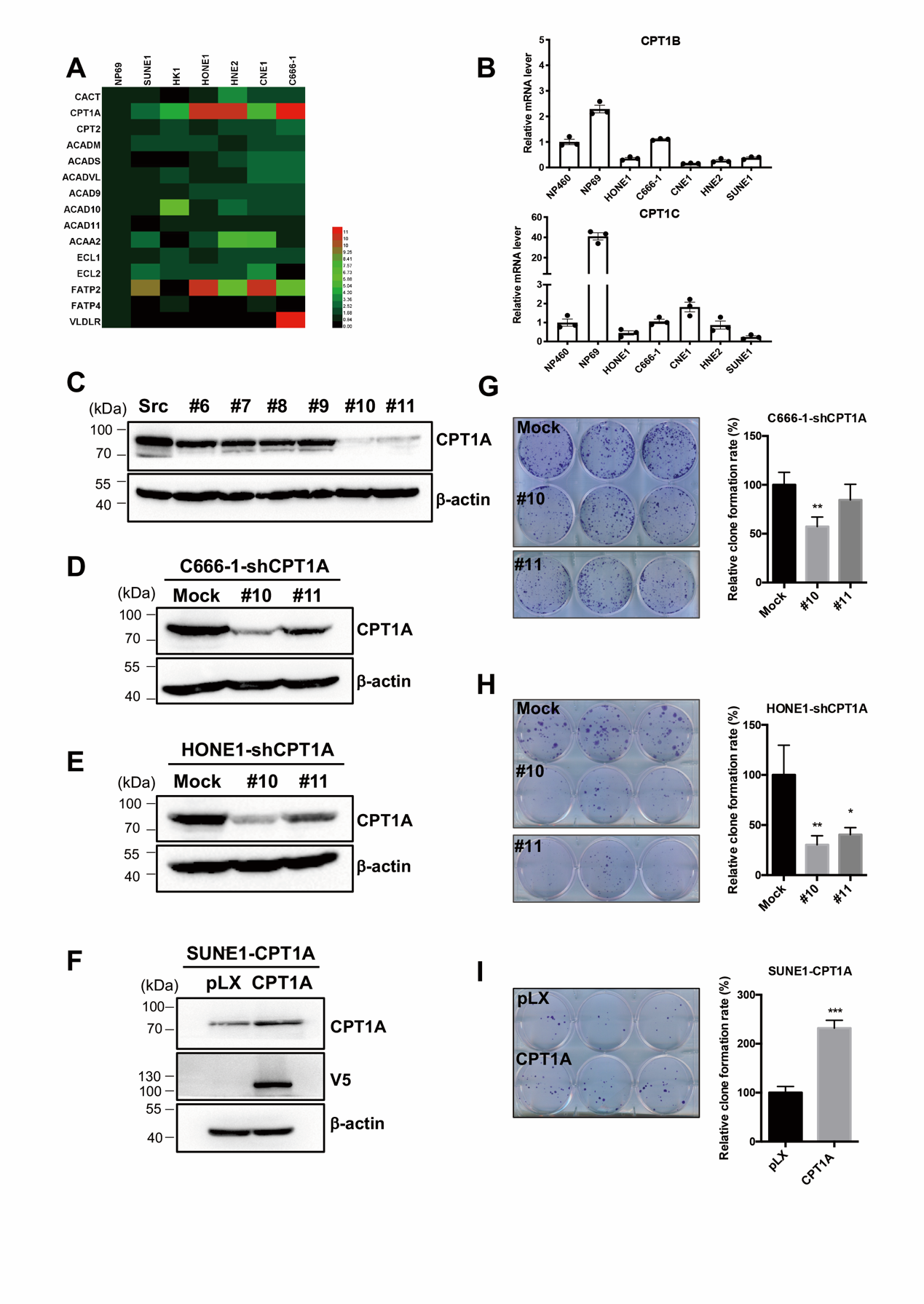


**Figure S1.** **Screening for effective targeting of shRNA, detection of knockdown and overexpression.**

**A** Heat map array detected by PCR showing fatty acid oxidation of beta genes in immortalized nasopharyngeal epithelial cells (NP69) and nasopharyngeal carcinoma cell lines (SUNE1, HK1, HONE1, HNE2, CNE1, C666-1).

**B** Real-time PCR was performed to examine CPT1B and CPT1C expression in the indicated cells.

**C** Analysis of the blocking effectiveness of 6 shRNA sequences from the RNAi Consortium (TRC, Broad Institute) and identification of two CPT1A shRNAs having on-target knockdown effects.

**D-F** Knockdown of CPT1A expression in HONE1 and C666-1 cell lines by using lentivirus with the #10 and #11 sequences (**D** and **E**), overexpression of CPT1A in the SUNE1 cell line (**F**).

**G-I** Significant increase in colony formation compared with control-transfected cells (* p < 0.05, ** p < 0.01, *** p < 0.001), n ≥ 3.

**Figure S2**


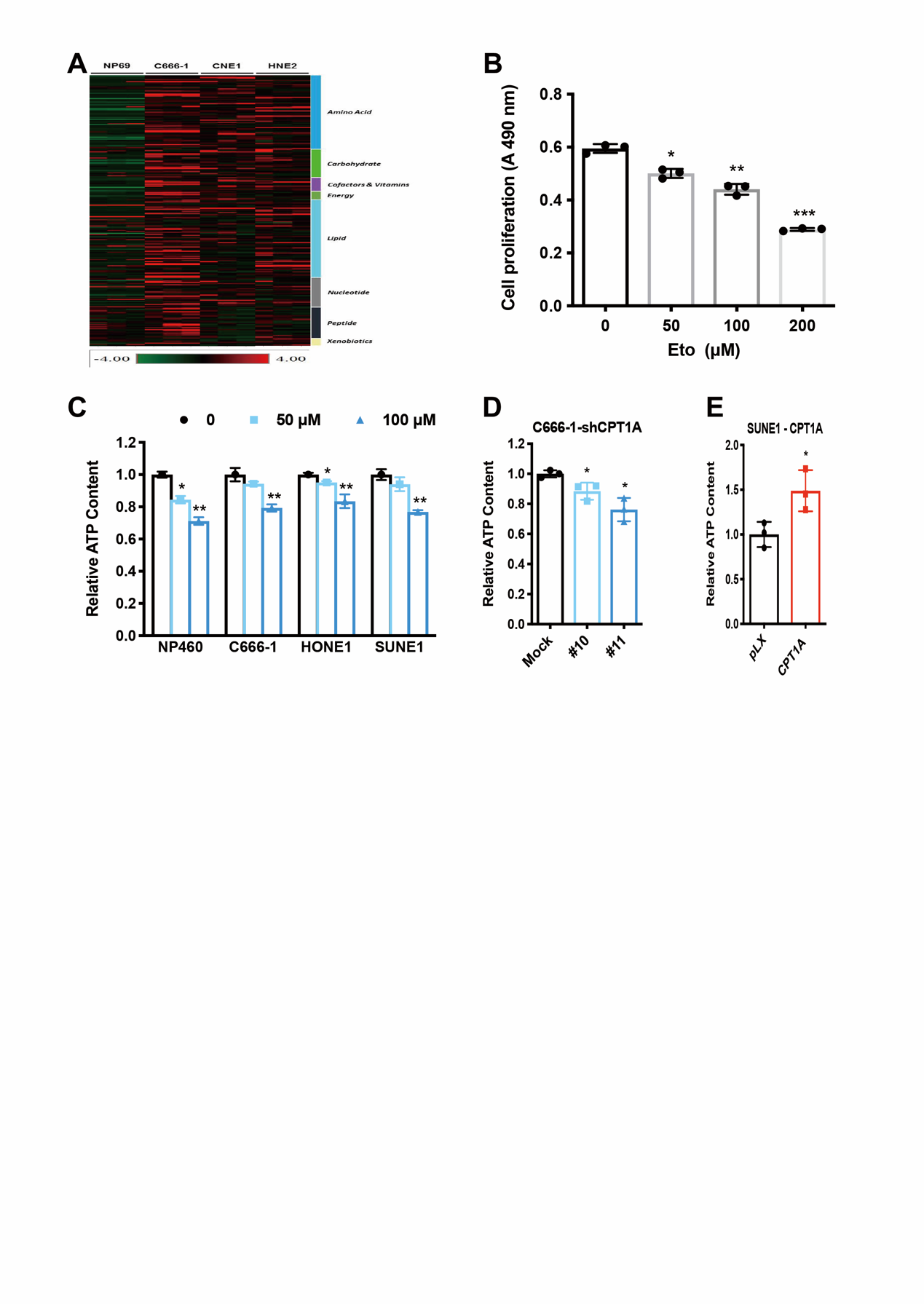


**Figure S2.** **CPT1A-promoted cell proliferation relies on FAO in nasopharyngeal carcinoma cells.**

**A** Heat map of 265 biochemical factors in lysates from three replicates each of immortalized nasopharyngeal epithelial cells (NP69) and NPC cells (C666-1, CNE1, and HNE2). The relative fold change for each factor in each sample is represented as the relative average increase (red) or decrease (green).

**B** MTS assay showing proliferation of C666-1 cells treated with different concentrations of Eto. Results are plotted as the mean absorbance at 490 nm ± SEM of 3 independent experiments (* p < 0.05, ** p < 0.01, *** p < 0.001).

**C** ATP levels of the groups of indicated cells with different concentrations of Eto treatment for 24 h. Data are mean ± SEM (* p < 0.05, ** p < 0.01), n ≥ 3.

**D, E** CPT1A increases the ATP content in nasopharyngeal carcinoma cells. Knocking down CPT1A in C666-1 impairs the ATP content in NPC cells (**D**); overexpression of CPT1A in SUNE1 cells raises the ATP content compared with the control (**E**). Data are mean ± SEM (* p < 0.05), n ≥ 3.

**Figure S3**

**
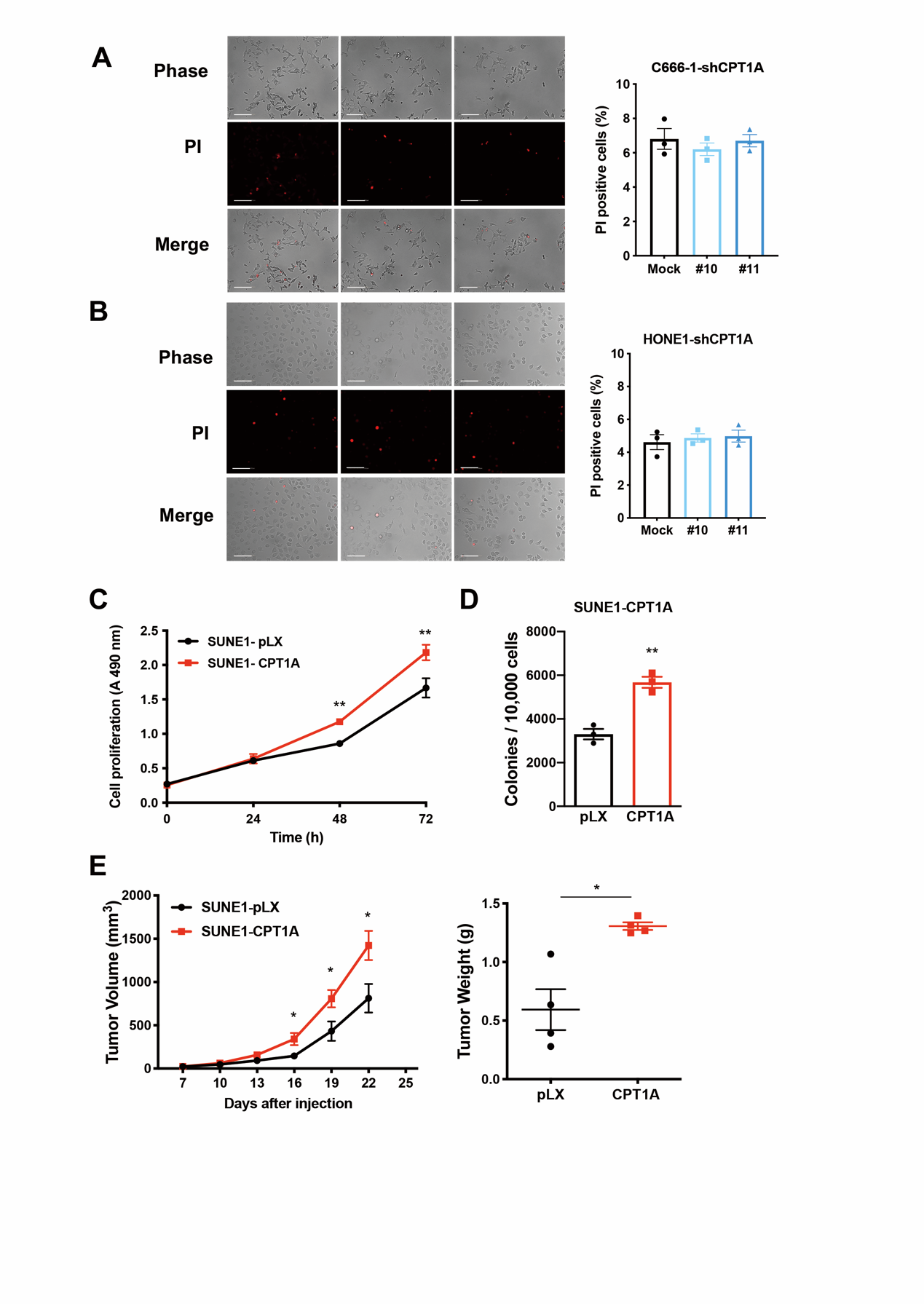
**

**Figure S3. Differential expression of CPT1A affects tumorigenic properties of NPC cells.**

**A, B** Left, representative images of staining with PI (red) in C666-1-shCPT1A (**A**) or HONE1-shCPT1A cells (**B**). (Scale bar, 100 μm). Right, statistical analyses of cells positive stained for PI. Data are mean ± SEM, n ≥ 3.

**C** MTS assay was performed to evaluate the effect of CPT1A on proliferation in SUNE1-CPT1A cells. Data are mean ± SEM (** p < 0.01), n ≥ 3.

**D** Colony formation assay in SUNE1-CPT1A cell. Data are mean ± SEM (** p < 0.01), n ≥ 3.

**E** Overexpression of CPT1A attenuates tumorigenity in SUNE1 cells. Left, tumor growth curve of mice injected with SUNE1-CPT1A cells. Right, tumor weights from each group are shown. Dots represent individual mice (n=4 per group). Error bars represent mean ± SEM (* p < 0.05).

**Figure S4**


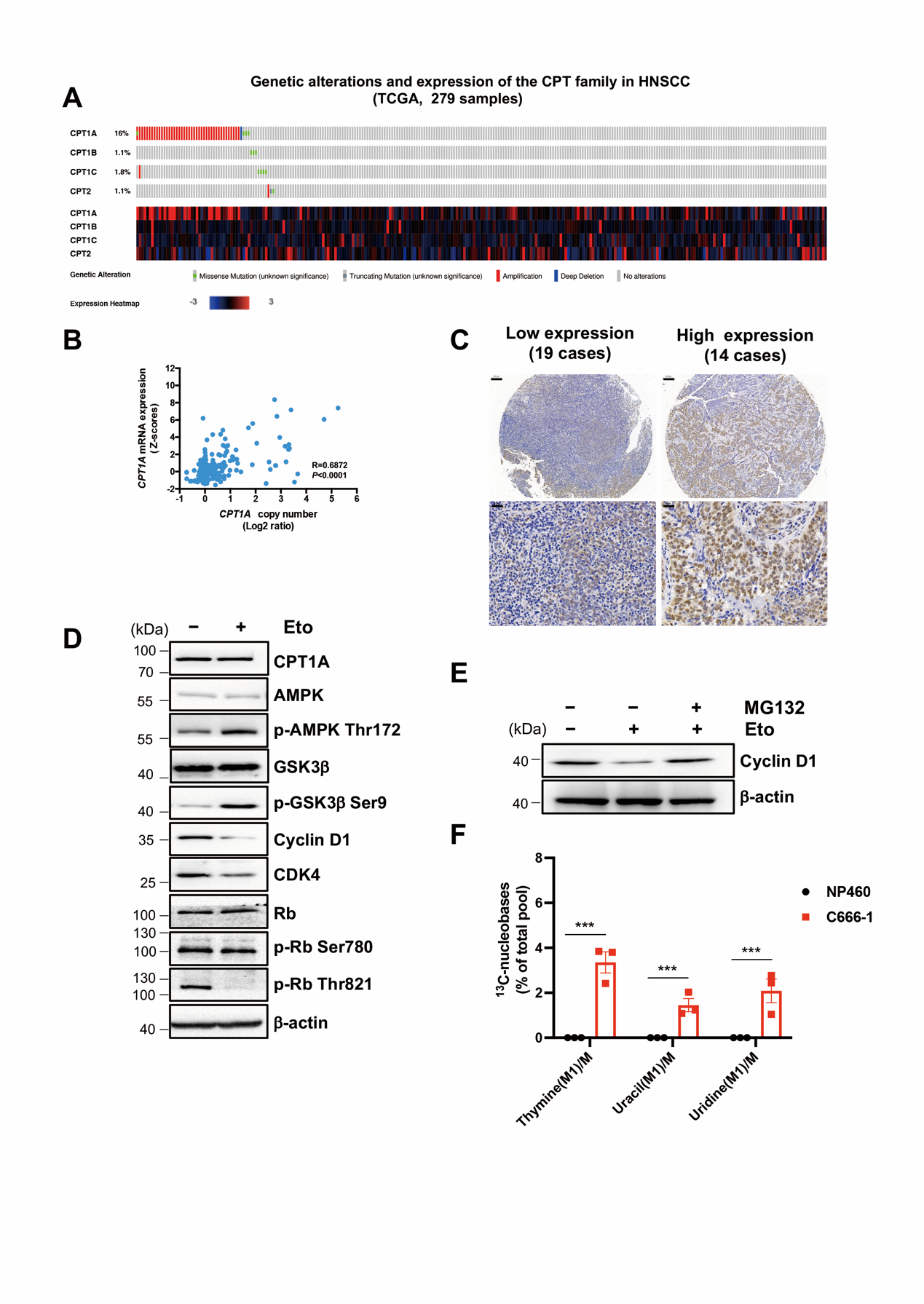


**Figure S4. CPT1A is abnormally activated in nasopharyngeal carcinoma.**

**A** Oncoprint (cBioportal) for the HNSCC TCGA provisional cohort of tumors with copy number alterations, showing tumors with *CPT* family gene amplifications. Percentages are relative to the complete number of tumors in the cohort. Data were extracted from the TCGA data set of HNSCC within the cBioPortal database. Analysis of genomic alterations in the two genes in the TCGA cohort (n = 279).

**B** Scatterplots of *CPT1A* copy number versus messenger RNA expression in HNSCC (n = 279).

**C** Representative IHC staining of CPT1A expression from the tissue microarray (NPC 961) of nasopharyngeal squamous cell carcinoma patients. (Scale bar, 100 μm; scale bar, 20 μm).

**D** Western blot analysis of Eto-targeting fatty acid oxidation down regulates the expression of G1 phase markers in HONE1 cells. β-actin was used as a control to confirm equal loading of protein.

**E** Eto-induced proteasome degradation of cyclin D1 was evaluated by Western blotting in C666-1 cells. The cells were pretreated with MG-132 (10 μM) for 6 h, or after pretreatment, Eto (100 μM) was added for an additional 24 h. β-actin was used as a control to confirm the equal loading of protein.

**F** Labeling incorporation from ^13^C-palmitate into nucleobases in NP460 and C666-1 cells. Data are shown as percentage of ^13^C-thymine, ^13^C-uracil or ^13^C-uridine compared to the total pool of each corresponding nucleobase (*** p < 0.001).

**Figure S5**

**
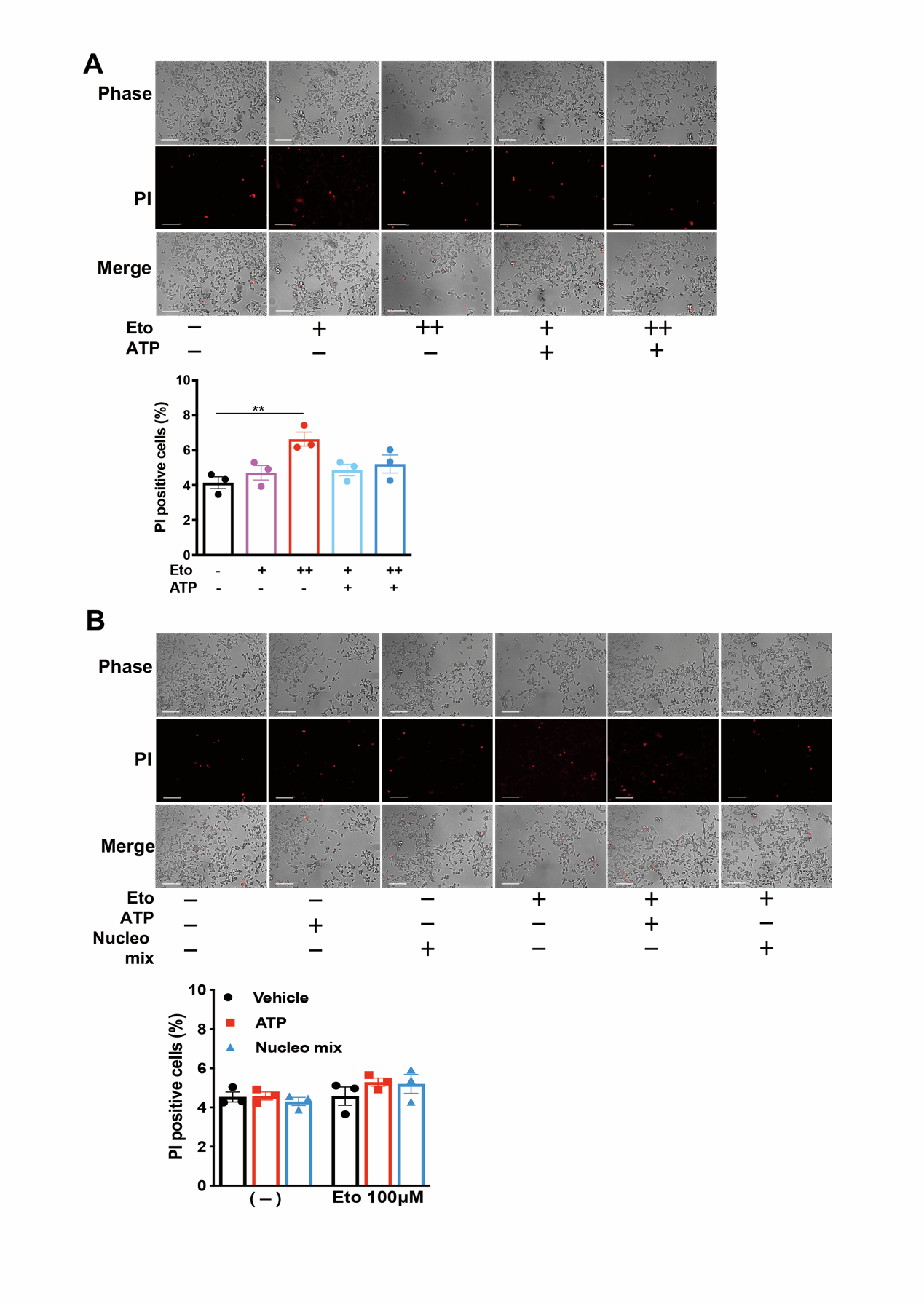
**

**Figure S5. Addition of ATP or nucleoside mix does not affect cell morphology and death.**

**A** Representative images of staining with PI (red) in C666-1 cells treated with different concentrations of Eto (100 μM or 200 μM) and ATP (50 μM). (Scale bar, 100 μm). Statistical analyses of cells positive stained for PI. Data are mean ± SEM (** p < 0.01), n ≥ 3.

**B** Representative images of staining with PI (red) in C666-1 cells treated with Eto and ATP (50 μM) or a nucleo mix (25 μM). (Scale bar, 100 μm). Statistical analyses of cells positive stained for PI. Data are mean ± SEM, n ≥ 3.
