# Supplementary figures and images for: CPT1A-mediated fatty acid oxidation promotes cell proliferation via nucleoside metabolism in nasopharyngeal carcinoma
Source: Cell Death Dis. 2022 Apr 11;13(4):331. doi: 10.1038/s41419-022-04730-y (PMC9001659; doi:10.1038/s41419-022-04730-y)

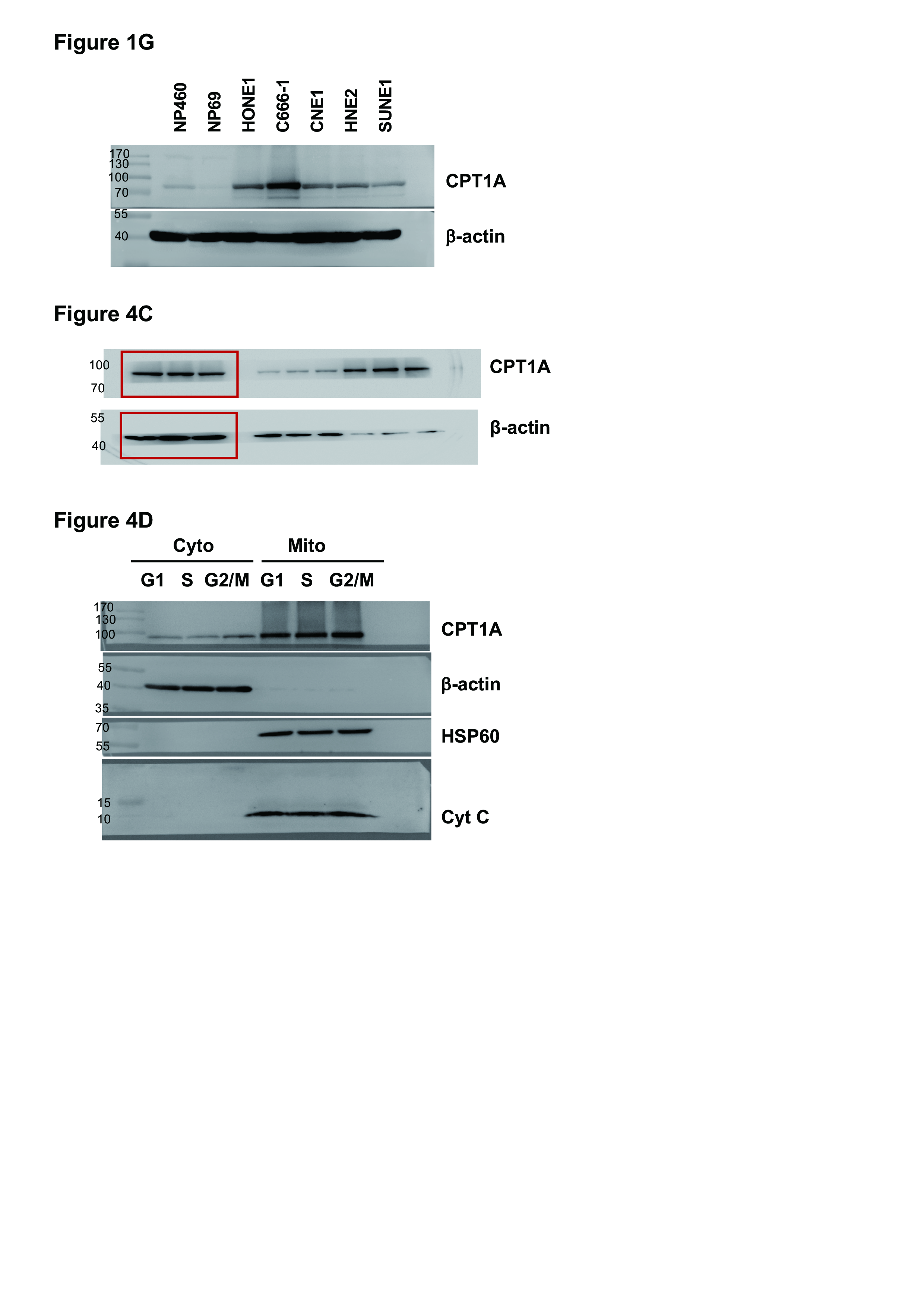

Supplement: Supplementary file 2 — Original Blot 1 [file 41419_2022_4730_MOESM2_ESM.tif]

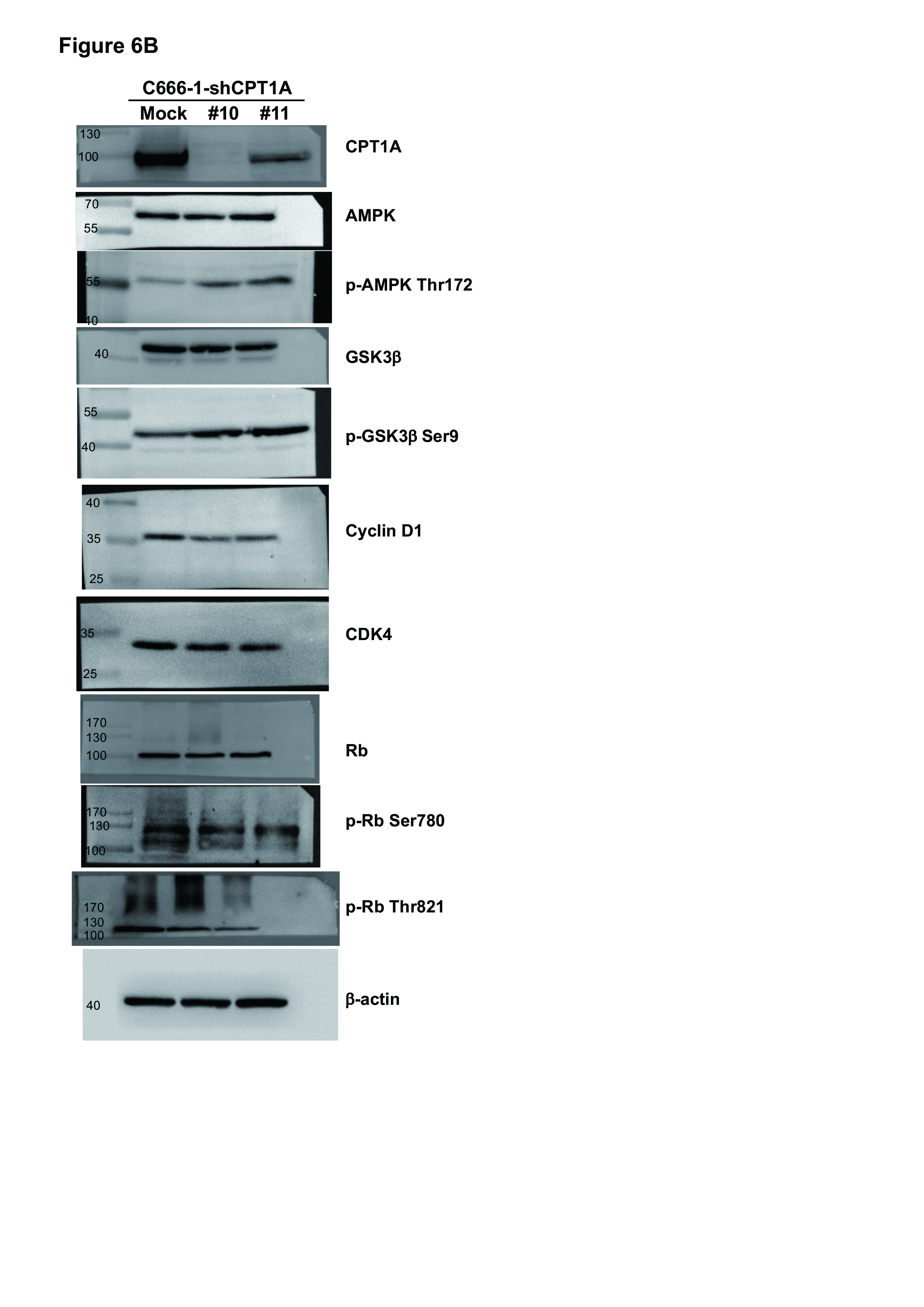

Supplement: Supplementary file 3 — Original Blot 2 [file 41419_2022_4730_MOESM3_ESM.tif]

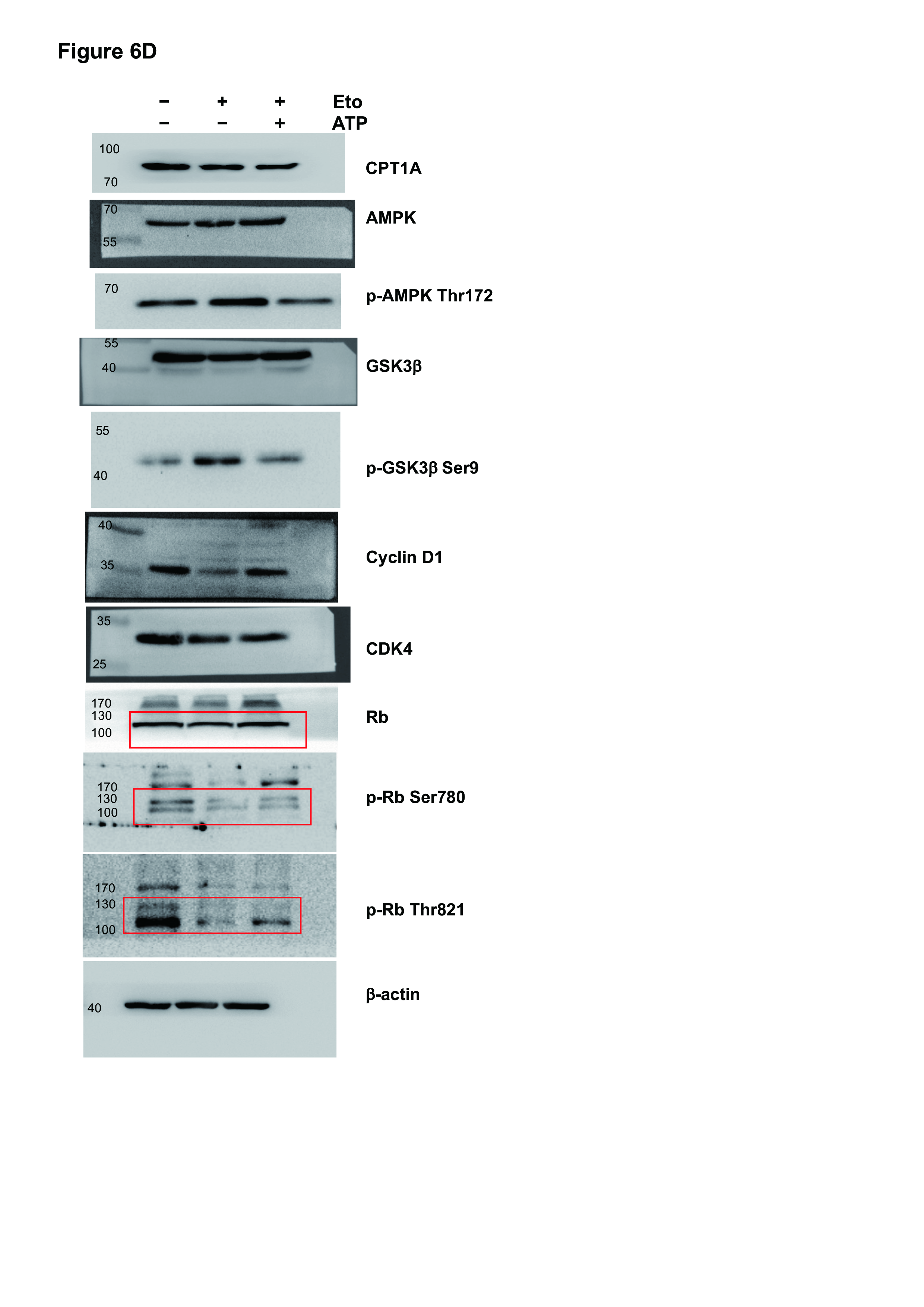

Supplement: Supplementary file 4 — Original Blot 3 [file 41419_2022_4730_MOESM4_ESM.tif]

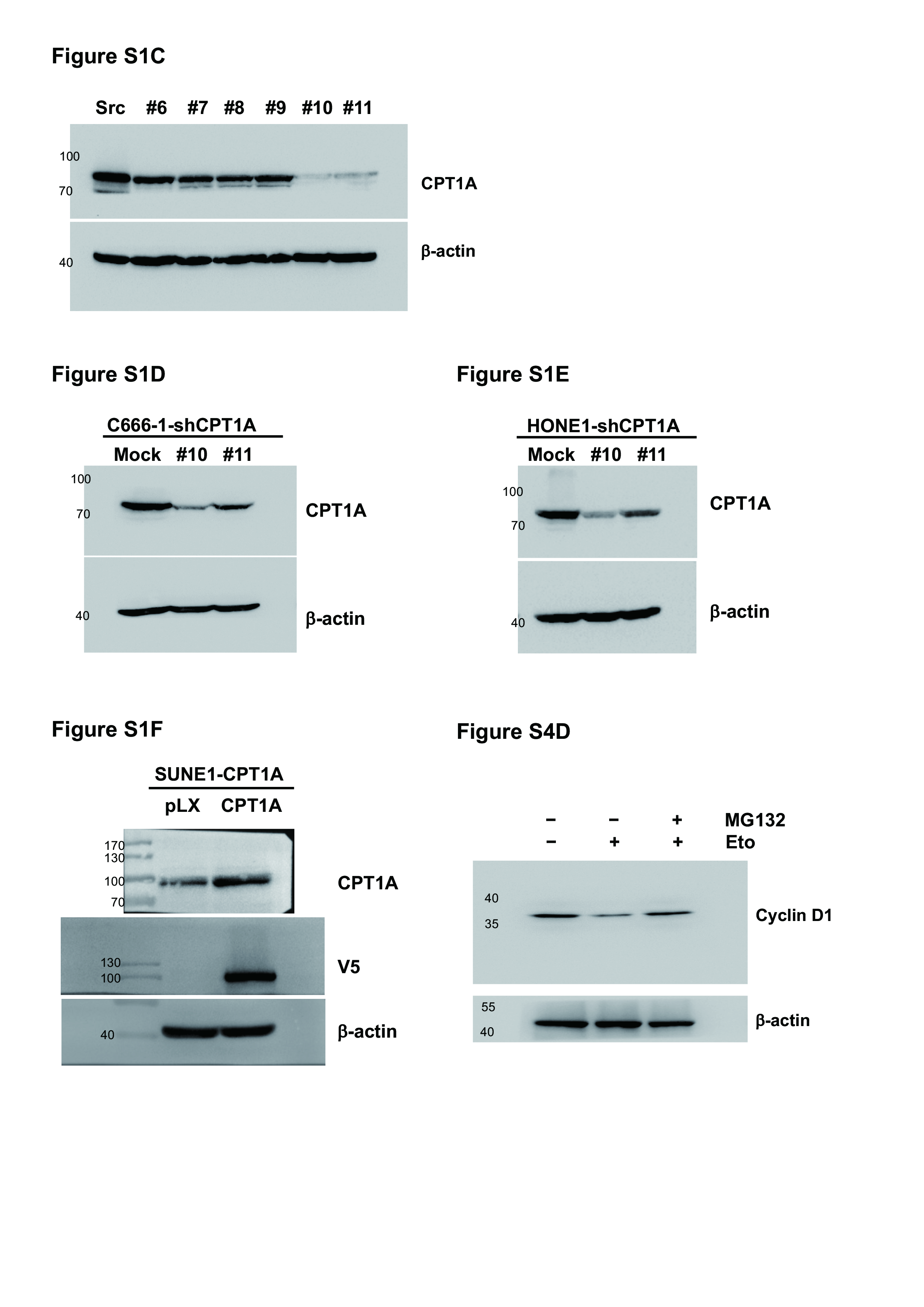

Supplement: Supplementary file 5 — Original Blot 4 [file 41419_2022_4730_MOESM5_ESM.tif]

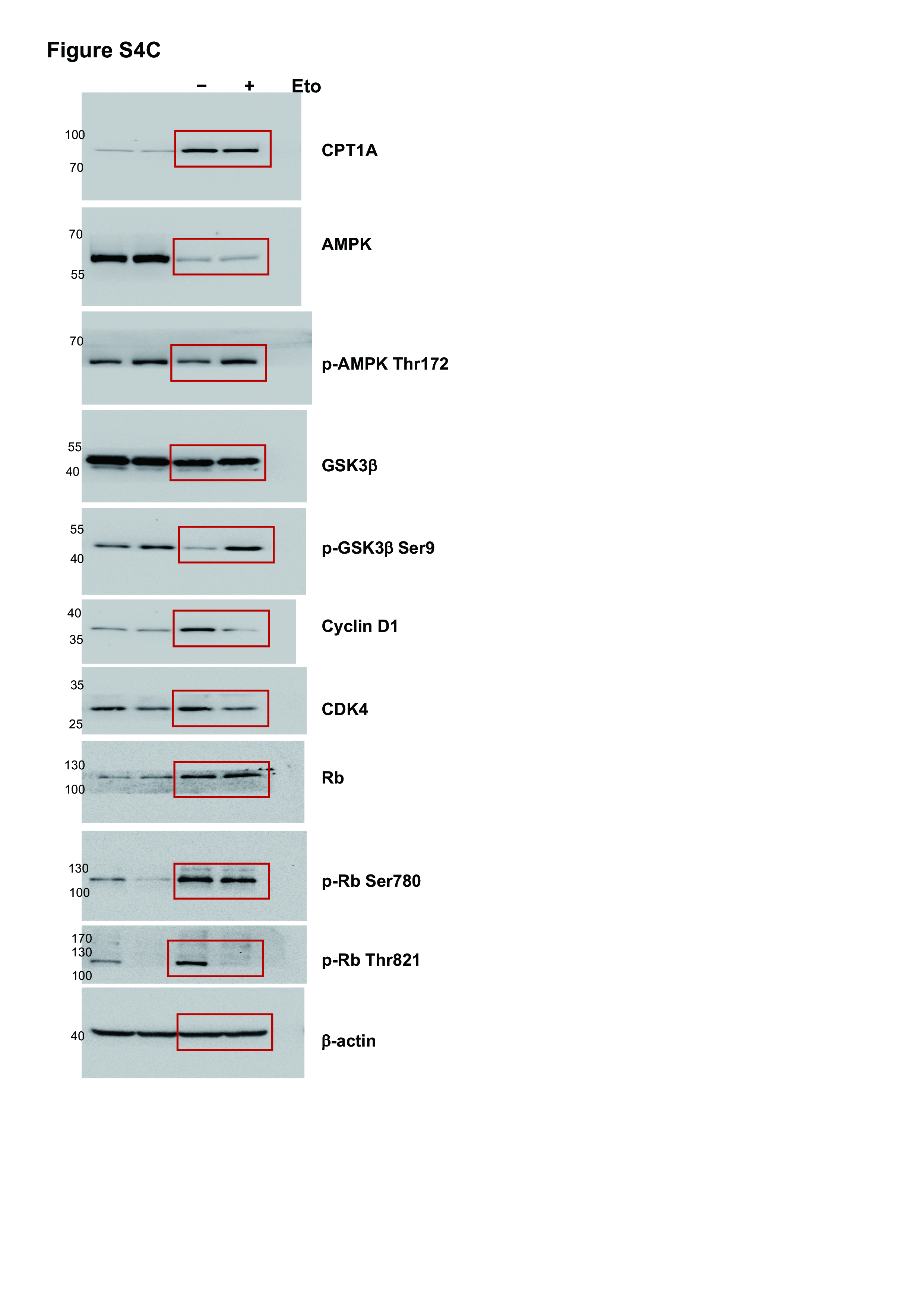

Supplement: Supplementary file 6 — Original Blot 5 [file 41419_2022_4730_MOESM6_ESM.tif]
